# The epigenetic reader PHF21B modulates murine social memory and synaptic plasticity–related genes

**DOI:** 10.1172/jci.insight.158081

**Published:** 2022-07-22

**Authors:** Eunice W.M. Chin, Qi Ma, Hongyu Ruan, Camille Chin, Aditya Somasundaram, Chunling Zhang, Chunyu Liu, Martin D. Lewis, Melissa White, Tracey L. Smith, Malcolm Battersby, Wei-Dong Yao, Xin-Yun Lu, Wadih Arap, Julio Licinio, Ma-Li Wong

**Affiliations:** 1 Department of Psychiatry and Behavioral Sciences,; 2 MD Degree Program, and; 3 Department of Neuroscience & Physiology, Norton College of Medicine, State University of New York Upstate Medical University, Syracuse, New York, USA.; 4 Neuropsychiatric Laboratory, Lifelong Health Research Unit, and; 5 Gene Editing Research Unit, South Australian Health and Medical Research Institute, Adelaide, South Australia, Australia.; 6 SA Genome Editing Facility, University of Adelaide, Adelaide, South Australia, Australia.; 7 Rutgers Cancer Institute of New Jersey, Newark, New Jersey, USA.; 8 Division of Cancer Biology, Department of Radiation Oncology, Rutgers New Jersey Medical School, Newark, New Jersey, USA.; 9 College of Medicine and Public Health, Flinders University, Bedford Park, South Australia, Australia.; 10 Department of Neuroscience & Regenerative Medicine, Medical College of Georgia at Augusta University, Augusta, Georgia, USA.; 11 Division of Hematology/Oncology, Department of Medicine, Rutgers New Jersey Medical School, Newark, New Jersey, USA.

**Keywords:** Neuroscience, Behavior, Epigenetics, Psychiatric diseases

## Abstract

Synaptic dysfunction is a manifestation of several neurobehavioral and neurological disorders. A major therapeutic challenge lies in uncovering the upstream regulatory factors controlling synaptic processes. Plant homeodomain (PHD) finger proteins are epigenetic readers whose dysfunctions are implicated in neurological disorders. However, the molecular mechanisms linking PHD protein deficits to disease remain unclear. Here, we generated a PHD finger protein 21B–depleted (Phf21b-depleted) mutant CRISPR mouse model (hereafter called Phf21b^Δ4/Δ4^) to examine Phf21b’s roles in the brain. Phf21b^Δ4/Δ4^ animals exhibited impaired social memory. In addition, reduced expression of synaptic proteins and impaired long-term potentiation were observed in the Phf21b^Δ4/Δ4^ hippocampi. Transcriptome profiling revealed differential expression of genes involved in synaptic plasticity processes. Furthermore, we characterized a potentially novel interaction of PHF21B with histone H3 trimethylated lysine 36 (H3K36me3), a histone modification associated with transcriptional activation, and the transcriptional factor CREB. These results establish PHF21B as an important upstream regulator of synaptic plasticity–related genes and a candidate therapeutic target for neurobehavioral dysfunction in mice, with potential applications in human neurological and psychiatric disorders.

## Graphical abstract

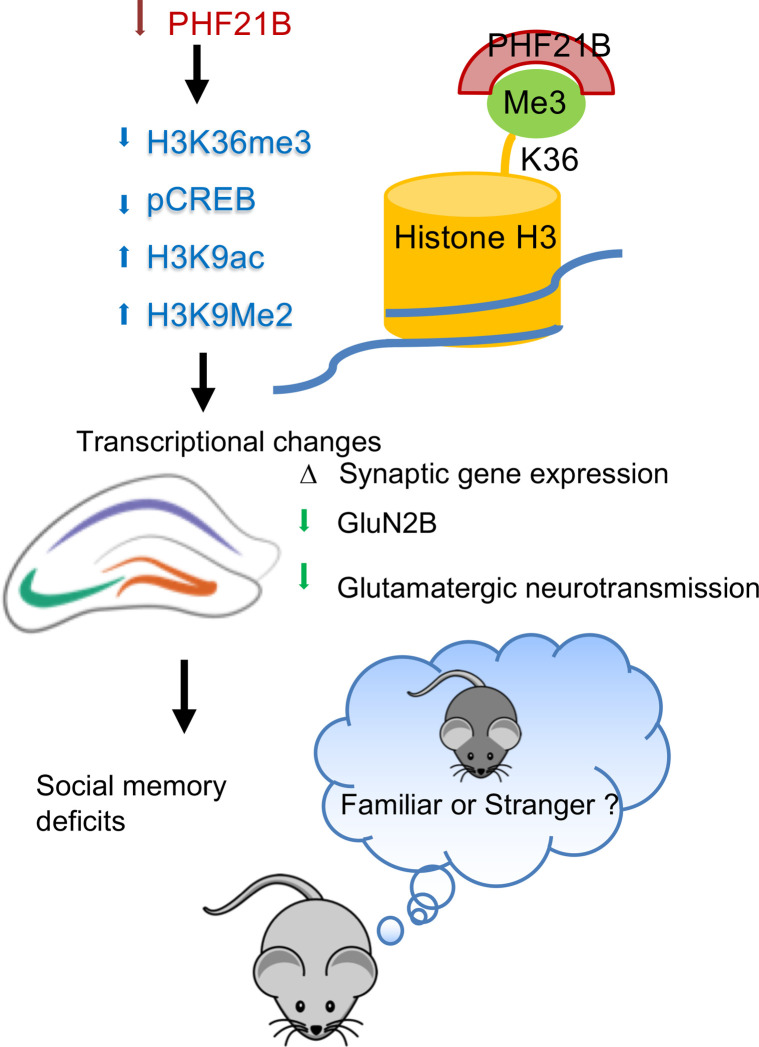

## Introduction

The formation of synaptic connections in the mammalian brain is a highly orchestrated process that involves precise control of gene expression by an assortment of integrated epigenetic regulators and transcriptional factors. Misregulation of synaptic development, function, and plasticity gives rise to defective neuronal circuitry, resulting in numerous neurobehavioral disorders (1, 2). Synaptic dysfunction is a common denominator of neurodevelopmental disorders, such as autism spectrum disorder, intellectual disability, and schizophrenia, and neurodegenerative diseases, such as Alzheimer’s, Parkinson’s, and Huntington’s (2–4). For instance, NMDA receptor–mediated (NMDAR-mediated) and α-amino-3-hydroxy-5-methyl-4-isoxazolepropionic acid receptor–mediated (AMPAR-mediated) transmission is adversely affected in autism spectrum disorder and Alzheimer’s disease. In addition, mutations in the genes encoding postsynaptic scaffolding protein SH3 and multiple ankyrin repeat domains (SHANK) family members are risk factors for autism and schizophrenia (1). Moreover, whole-exome sequencing and targeted candidate gene sequencing of patient cohorts and family pedigree studies have identified rare variants and genetic risk factors associated with neurobehavioral disorders (2). Although these studies allow for understanding the downstream proteins involved in synaptic dysregulation, much is still unknown about the upstream regulatory factors and molecular mechanisms controlling their expression.

The plant homeodomain (PHD) finger proteins are important epigenetic readers that are associated with chromatin, regulating gene activities. Interactions between 2 subclasses of PHD fingers and histone 3 trimethylated lysine 4 (H3K4me3) and unmodified H3K4 (H3K4me0) have been reported (5, 6). Germline mutations and translocations of the PHD finger in proteins are implicated in certain neurological disorders (e.g., NSD1 and ATRX) (7–10), cancer (e.g., ING1 and PHF23) (11–13), and other immunological disorders (e.g., RAG2 and AIRE) (14–17). A recent study found the PHD finger protein 21B (PHF21B) to be highly expressed in the neurogenic phase of cortical development. Its depletion resulted in the retention of neural progenitor cells in the proliferative zones (18). This finding is interesting because we have previously reported an association between rare single nucleotide variations near the *PHF21B* gene and increased major depressive disorder risk (19). Moreover, the human *PHF21B* gene locus resides in the chromosome 22q13.3 region, whose deletion has been established to be linked to the neurodevelopmental disorder Phelan-McDermid syndrome (20). Synaptogenesis is a major process in cortical development, and synaptic dysfunction manifests in neurobehavioral disorders; therefore, we hypothesized that PHF21B regulates the expression of synaptic plasticity–related genes.

This study generated a PHF21B-depleted mouse model that exhibits social memory deficits. We further identified PHF21B as an important modulator of synaptic transmission and characterized its mechanistic role in interacting with the transcriptional factor CREB and regulating transcriptional activation of synaptic genes. These findings elucidate what we believe are previously unrecognized functional roles for PHF21B in the mammalian brain, with potential implications for several human neurological diseases.

## Results

### Design, generation, and verification of Phf21b^Δ4/Δ4^ animals.

**Figure 1:**
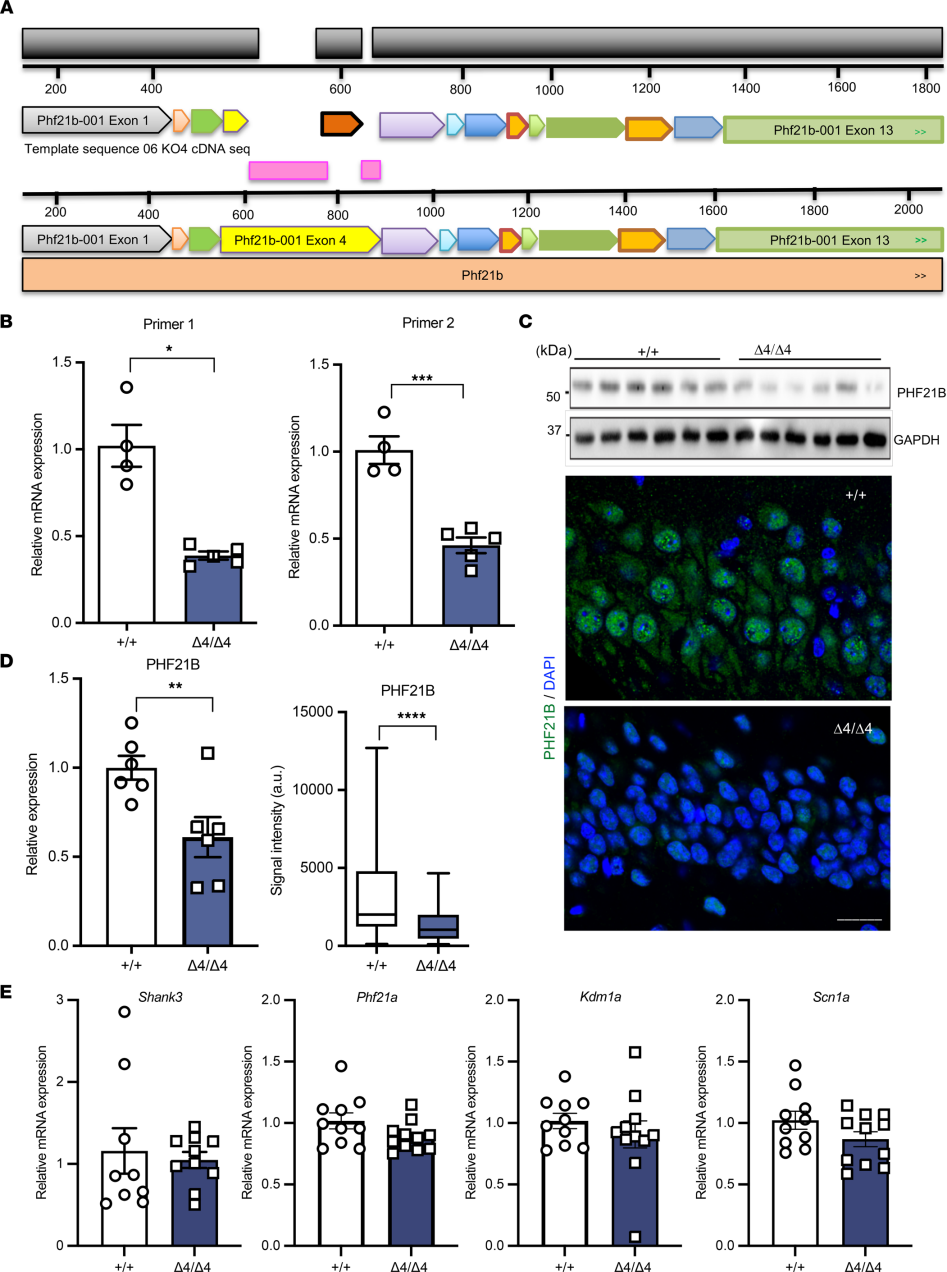
Generation and verification of Phf21b^Δ4/Δ4^ animals. (**A**) Alignment of PHF21B cDNA sequences in Phf21b^+/+^ (+/+) and Phf21b^Δ4/Δ4^ (Δ4/Δ4) mice. Pink regions depict the sequences that were deleted in the Δ4/Δ4 mouse model. (**B**) Quantification of *Phf21b* mRNA transcript expression in +/+ and Δ4/Δ4 hippocampal tissues by using 2 different targeting primer pairs; *n* = 4–5/group. (**C**) Representative images of (top panels) hippocampal Western blots (each lane is an individual animal) and (bottom panels) immunostained nuclei of CA1 neurons of PHF21B expression levels in +/+ and Δ4/Δ4 animals; scale bar: 20 μm. (**D**) Quantification of PHF21B immunoblot (left, *n* = 6/group) and signal intensity in immunostained (right, *n* = 5/group) +/+ and Δ4/Δ4 hippocampal sections. (**E**) mRNA transcript expression of (from left to right) *Shank3*, *Phf21a*, *Kdm1a*, and *Scn1a* in +/+ and Δ4/Δ4 animals, evaluated once; *n* = 10/group. Values are presented as mean ± SEM (**B**, **D** left graph, and **E**) or minimum to maximum and line at the median (**D** right graph); Student’s *t* test/Mann-Whitney test; * *P* < 0.05; ** *P* < 0.01; *** *P* < 0.001; **** *P* < 0.0001.

In order to investigate the functional roles of PHF21B in the brain, we generated a whole-body *Phf21b*-depleted mouse line (Phf21b^Δ4/Δ4^) by using CRISPR/Cas9 technology. We specifically targeted exon 4 of the *Phf21b* gene, containing its corresponding PHD domain (Figure 1A). A 268 bp deletion in exon 4 was generated, resulting in a missense out-of-frame mutation and decreasing *Phf21b* expression, verified at the transcript (Figure 1B) and protein (Figure 1, C and D) levels. The animals expressed approximately 60% less PHF21B than their WT counterparts (Figure 1, B–D). Off-target analysis of the CRISPR-mediated editing of *Phf21b* was validated via PCR (Supplemental Figure 1, A–C, and Supplemental Table 1; supplemental material available online with this article; https://doi.org/10.1172/jci.insight.158081DS1). Further, the transcript level of *Shank3*, whose locus (15E3) in the mouse chromosome is near the *Phf21b* locus (15E2), showed no detectable expression level changes (Figure 1E). In addition, no detectable differences were found in the transcript levels of *Phf21a*, a close paralog of *Phf21b*, and the PHF21A downstream targets *Kdm1a* and *Scn1a* (Figure 1E). PHF21B was highly expressed in neural tissue, including the cortex, hippocampus, cerebellum, and spinal cord, compared with other peripheral tissues (Supplemental Figure 1D); however, adult Phf21b^+/+^ and Phf21b^Δ4/Δ4^ animals were not observed to differ in general health or physical measures.

### Phf21b^Δ4/Δ4^ mice exhibit social memory deficits.

**Figure 2:**
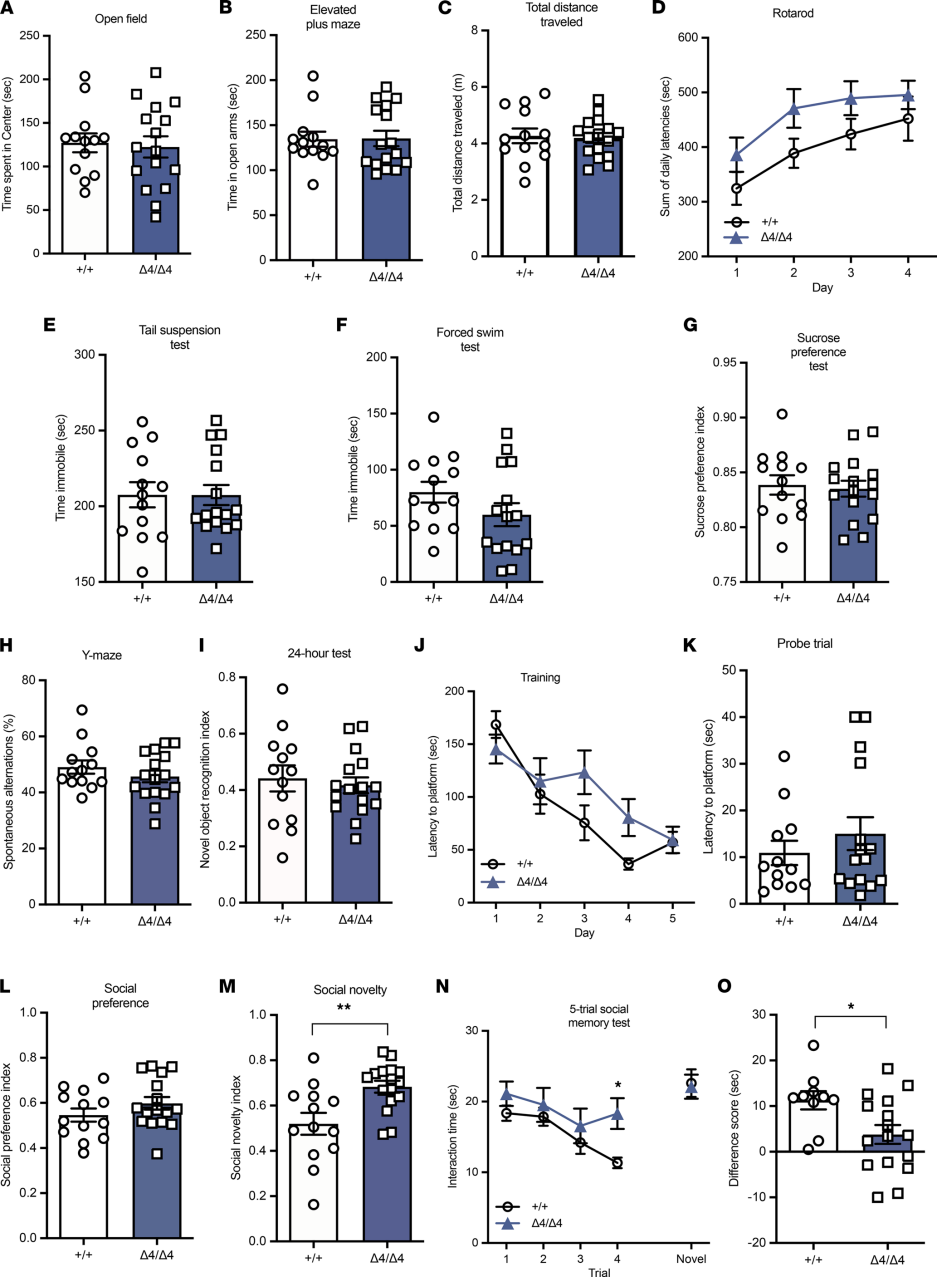
Phf21b^Δ4/Δ4^ animals exhibit social memory deficits. Assays characterizing the behavioral phenotype of the Phf21b^+/+^ (+/+) and Phf21b^Δ4/Δ4^ (Δ4/Δ4) mice. (**A**) Time spent in the center of the open field arena. (**B**) Time spent in the open arms of the elevated-plus maze. (**C**) Total distance traveled in the elevated-plus maze. (**D**) Sum of latencies to fall from an accelerating rotarod as a motor coordination assessment in the +/+ and Δ4/Δ4 mice. (**E**) Time spent immobile in the tail suspension and (**F**) forced swim tests. (**G**) Sucrose preference index as a measure of anhedonia. (**H**) Percentage of spontaneous alternations in the Y-maze test for spatial working memory. (**I**) Novel object recognition index of the +/+ and Δ4/Δ4 animals in a test trial 24 hours posttraining. (**J**) Daily latencies to platform over 5 training days in the Morris water maze. (**K**) Latency to platform location during the probe test on the sixth day of the Morris water maze test. (**L**) Preference for social interaction of the +/+ and Δ4/Δ4 animals. (**M**) Preference for social novelty of the +/+ and Δ4/Δ4 mice. (**N**) Interaction time between a subject animal with the same stranger animal over 4 trials, with an intertrial interval of 10 minutes. A novel stranger was introduced at the fifth trial. (**O**) The difference score is given by the difference in interaction time between the fifth (dishabituation) trial with the novel stranger and the final/fourth (habituation) trial with the familiar stranger. Values are presented as mean ± SEM; *n* = 13–16/group; Student’s *t* test/Mann-Whitney test (**A**–**C**, **E**–**I**, **K**–**M**, and **O**); 2-way ANOVA (**D**, **J**, and **N**); * *P* < 0.05; ** *P* < 0.01.

We assessed the behavioral phenotype of the Phf21b^Δ4/Δ4^ animals by subjecting them to a series of behavioral tests. The Phf21b^Δ4/Δ4^ mice did not exhibit anxiety-like behaviors, as the amount of time they spent in the center of the open field (Figure 2A) and the open arms of the elevated plus-maze (Figure 2B and Supplemental Figure 2A) did not differ from that of their WT littermates. The Phf21b^Δ4/Δ4^ animals also had normal locomotor activity, as assessed by the total distance traveled in the elevated plus maze (Figure 2C) and the rotarod test (Figure 2D). Moreover, the Phf21b^Δ4/Δ4^ mice did not exhibit behavior despair, as they did not have increased immobility times in the tail suspension (Figure 2E) and forced swim (Figure 2F) tests compared to Phf21b^+/+^ animals. They also did not display anhedonia, as they had similar levels of sucrose preference (Figure 2G) to WT mice.

The Phf21b^Δ4/Δ4^ animals did not differ from their WT counterparts in the percentage of spontaneous alternations in the Y-maze (Figure 2H and Supplemental Figure 2B) and the recognition of a novel object (Figure 2I); these results indicate that they have normal working memory and long-term recognition memory. In addition, the Phf21b^Δ4/Δ4^ animals’ time to learn the location of the platform (Figure 2J), and the mean latency for the Phf21b^Δ4/Δ4^ mice to reach the platform location in the probe trial in the Morris water maze, did not differ from that of the WT animals (Figure 2K). These results suggest that the Phf21b^Δ4/Δ4^ mice have intact learning and long-term spatial reference memory.

Because the human PHF21B gene is located in a chromosomal region linked to autism spectrum disorders, we also tested the sociability of the animals. The social preference index of the Phf21b^Δ4/Δ4^ mice did not differ from the Phf21b^+/+^ animals (Figure 2L). However, the social novelty index of the Phf21b^Δ4/Δ4^ mice was significantly greater than that of the WT animals (Figure 2M), which suggests deficits in social memory. We then administered the 5-trial social memory test to probe this intriguing finding further. The Phf21b^Δ4/Δ4^ mice were able to recognize the novel stranger. However, they could not remember the familiar stranger they had been habituated with. There was no decrease in interaction time during the habituation trials for the Phf21b^Δ4/Δ4^ mice compared to the Phf21b^+/+^ animals (Figure 2N and Supplemental Figure 2, C and D). The score difference between the last habituation trial and the novel trial was significantly lower for the Phf21b^Δ4/Δ4^ mice than the Phf21b^+/+^ animals (Figure 2O). The Phf21b^+/+^ and Phf21b^Δ4/Δ4^ mice did not show disability in detecting or discriminating either nonsocial or social odors (Supplemental Figure 2E). These behavioral data suggest that decreased expression of PHF21B results in impaired social memory.

### Decreased PHF21B expression results in thinner cortices and reduced neurogenesis and astrocyte numbers.

**Figure 3:**
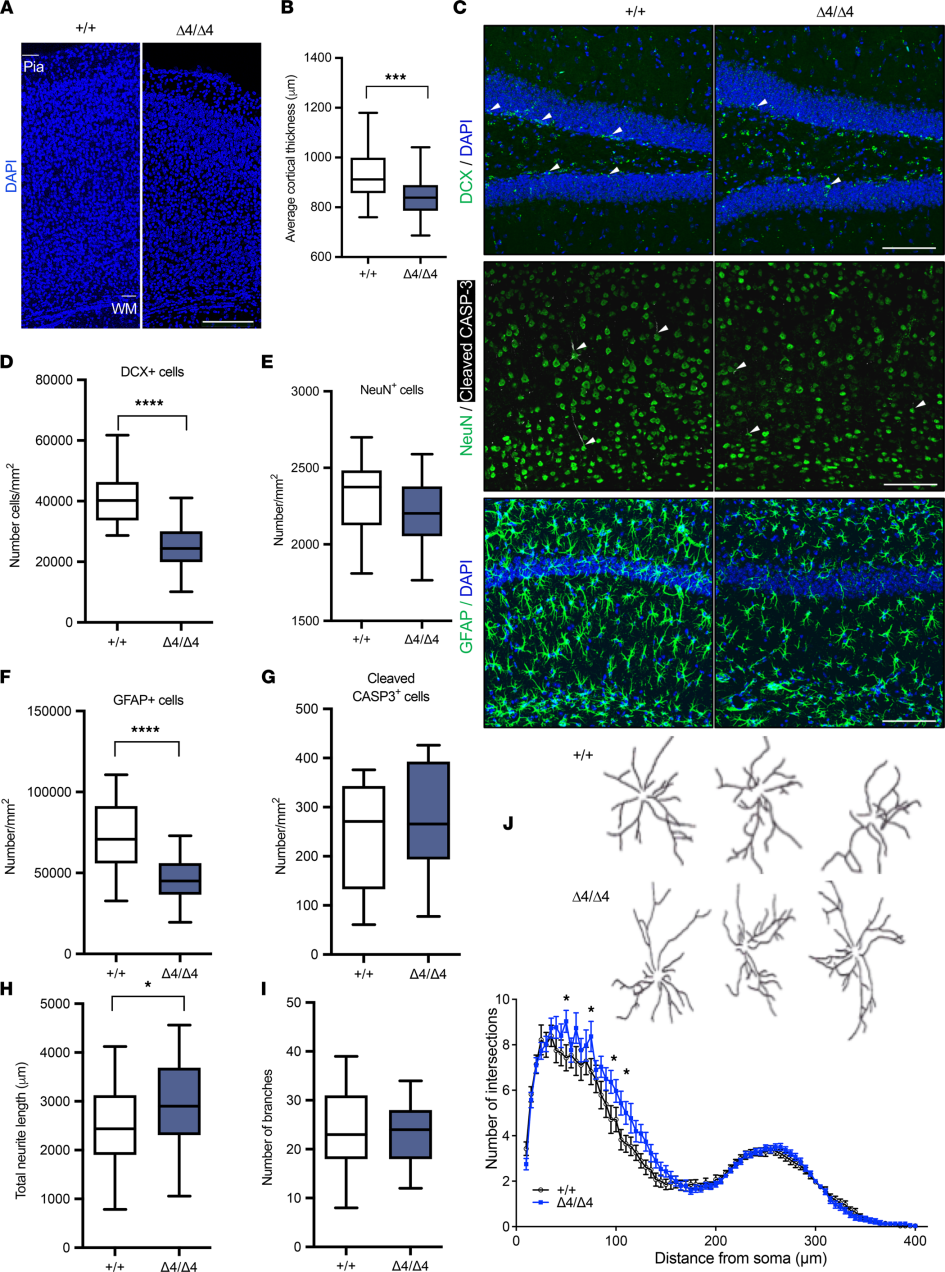
Decreased PHF21B expression results in thinner cortices and reduced neurogenesis and astrocyte numbers. (**A**) Representative images of the cortices from the pia to the white matter (WM) layers in the Phf21b^+/+^ (+/+) and Phf21b^Δ4/Δ4^ (Δ4/Δ4) mouse brains; scale bar: 200 μm. (**B**) Quantification of the average cortical thickness of the Phf21b^+/+^ and Phf21b^Δ4/Δ4^ brains; *n* = 5. (**C**) Representative images of (top panels) DCX-positive cells (white arrowheads) in the dentate gyrus, (middle panels) NEUN-positive cells and cleaved CASP-3–positive cells (white arrowheads) in the cortex, and (bottom panels) GFAP-positive astrocytes in the hippocampus, of +/+ and Δ4/Δ4 animals; scale bars: 100 μm. Number of (**D**) DCX-positive cells, (**E**) NEUN-positive cells, (**F**) GFAP-positive cells, and (**G**) cleaved CASP-3–positive cells in the brains of +/+ and Δ4/Δ4 mice, *n* = 3–5/group. (**H**) Total neurite length, (**I**) number of branches of +/+ and Δ4/Δ4 primary hippocampal neurons, and (**J**) (top panels) representative tracings of +/+ and Δ4/Δ4 neurons and (bottom graph) Sholl analysis quantifying the complexity of dendritic arborization of +/+ and Δ4/Δ4 hippocampal neurons; *n* = 30 neurons from 3 independent cultures. Values are presented as mean ± SEM (**J**) or minimum to maximum and line at the median (**B** and **D**–**I**); Student’s *t* test/Mann-Whitney test (**B** and **D**–**I**) or mixed effects analysis; * *P* < 0.05; *** *P* < 0.001; **** *P* <0.0001.

We next examined the brains of the Phf21b^Δ4/Δ4^ mice, given their behavioral deficits. We found that the average cortical thickness of the Phf21b^Δ4/Δ4^ brain was significantly reduced by approximately 100 μm compared with the Phf21b^+/+^ brain (Figure 3, A and B). This result suggests reduced cell numbers and/or diminished neuronal structure. PHF21B modulates cell fate determination (18); therefore, we immunostained the brains for neurogenic markers. There was a reduction in the number of immature neurons (DCX-positive cells) in the dentate gyrus of the Phf21b^Δ4/Δ4^ brain compared with the Phf21b^+/+^ brain (Figure 3, C and D). No detectable difference was found in the number of mature neurons (NEUN-positive cells) (Figure 3, C and E).

In an attempt to account for the thinner cortices of the Phf21b^Δ4/Δ4^ brain, we examined the non-neuronal cell population and found decreased astrocyte numbers in the Phf21b^Δ4/Δ4^ tissues (Figure 3, C and F). The numbers of pro-apoptotic cells (cleaved caspase-3–positive cells) were similar for the Phf21b^Δ4/Δ4^ and Phf21b^+/+^ brains (Figure 3, C and G). We further observed differences in the dendritic arborizations of Phf21b^Δ4/Δ4^ versus Phf21b^+/+^ in primary hippocampal neuronal culture. The total neurite length of Phf21b^Δ4/Δ4^ neurons was significantly greater than that of Phf21b^+/+^ neurons (Figure 3H), with no difference in their total number of branches (Figure 3I). Slight differences in dendritic complexity were also found, with Phf21b^Δ4/Δ4^ neurons having increased complexity in neurites closer to their cell bodies (Figure 3J and Supplemental Table 2). We concluded that PHF21B deficiency negatively affects neuronal and astrocytic cell populations.

### Decreased synaptic protein expression, glutamatergic neurotransmission, and GluN2B/Grin2b levels in the Phf21b^Δ4/Δ4^ hippocampus.

**Figure 4:**
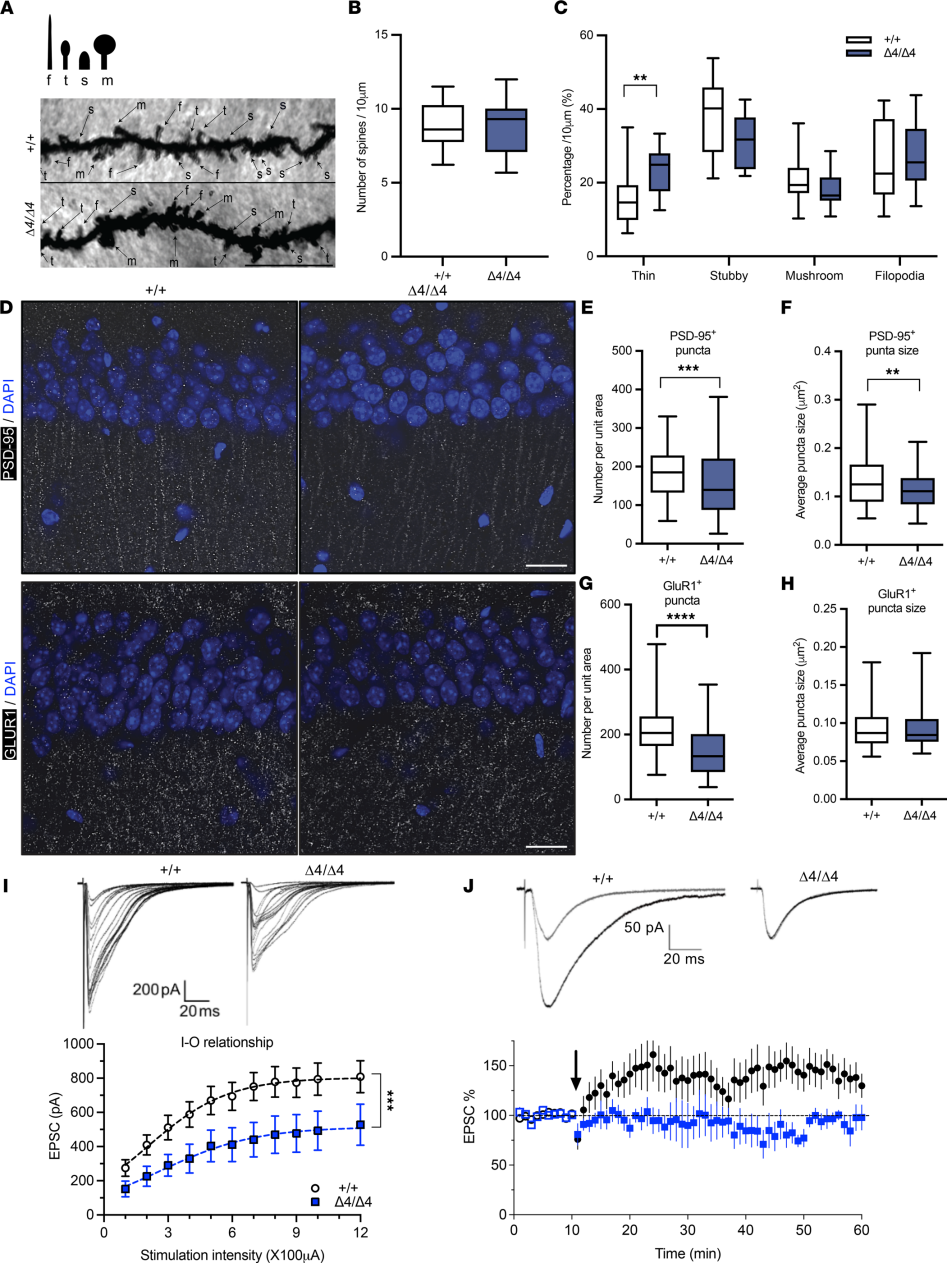
Decreased synaptic protein expression and glutamatergic neurotransmission in the Phf21b^Δ4/Δ4^ hippocampus. (**A**) (Top image) Dendritic spine shape classification; f, filopodia; t, thin, s, stubby; m, mushroom. (Bottom panels) Representative images of Golgi-stained neurites in Phf21b^+/+^ (+/+) and Phf21b^Δ4/Δ4^ (Δ4/Δ4) hippocampi; *n* = 3–5/group; scale bar: 10 μm. (**B**) Quantification of the number of dendritic spines per 10 μm. (**C**) Dendritic spines were classified according to their shapes, expressed as a percentage of the total number of spines per 10 μm of neurite, and compared in the +/+ and Δ4/Δ4 hippocampi. (**D**) Representative images of PSD-95–positive puncta and AMPAR subunit glutamate receptor 1–positive (GLUR1-positive) puncta in the CA1 region of the hippocampus of +/+ and Δ4/Δ4 mice; scale bars: 20 μm. (**E**) Number and (**F**) size of PSD-95-positive puncta in +/+ and Δ4/Δ4 hippocampi, *n* = 5. (**G**) Number and (**H**) size of GLUR1-positive puncta in +/+ and Δ4/Δ4 hippocampi; *n* = 5. (**I**) (Top images) Representative traces of (bottom graph) input-output (I/O) relationship of excitatory postsynaptic currents (EPSCs) recorded from CA1 pyramidal neurons in +/+ and Δ4/Δ4 hippocampi. Dotted lines are fitted to a semi-log equation: y = y0 + ym × log(*x*); *n* = 8–9 neurons. (**J**) (Top images) Representative traces of synaptic response before stimulation (gray trace) and after stimulation (black trace) in +/+ and Δ4/Δ4 CA1 neurons and (bottom graph) time course and magnitude of potentiation evoked by 240 stimuli at 4 Hz (black arrow); *n* = 7–11 neurons. Values are presented as mean ± SEM (**I** and **J**) or minimum to maximum and line at the median (**B**, **C**, and **E**–**H**); Student’s *t* test/Mann-Whitney test (**B**, **C**, and **E**–**H**), 2-way ANOVA (**C**), or mixed effects analysis (**I**); ** *P* < 0.01; *** *P* < 0.001; **** *P* < 0.0001.

The Cornus Ammonis (CA) 1 and CA2 regions, the principal pyramidal cell fields of the hippocampus, are frequently the focus of social memory research, and diminished astrocyte numbers suggest compromised synaptic maintenance (21). Thus, we next examined neuronal synapses in the Phf21b^Δ4/Δ4^ hippocampus via Golgi staining. Phf21b^Δ4/Δ4^ and Phf21b^+/+^ hippocampal neurons had similar numbers of dendritic spines per unit length (Figure 4, A and B). However, the Phf21b^Δ4/Δ4^ neurons had a larger proportion of thin spines than the Phf21b^+/+^ neurons (Figure 4C).

We also found that the Phf21b^Δ4/Δ4^ hippocampus expressed fewer postsynaptic density 95–positive (PSD-95–positive) clusters per unit area than the Phf21b^+/+^ hippocampus (Figure 4, D and E), and those clusters were smaller (Figure 4F). In addition, there were fewer AMPAR subunit GLUR1-expressing clusters in the hippocampal tissues of Phf21b^Δ4/Δ4^ mice (Figure 4, D, G, and H). These data suggest a compromised synaptic function. Indeed, we detected weaker glutamatergic synaptic transmission in ex vivo hippocampal slices of Phf21b^Δ4/Δ4^ mice compared with Phf21b^+/+^ mice, as described by the reduced input-output relationship recorded from the Phf21b^Δ4/Δ4^ CA1 neurons (Figure 4I). We did not notice differences in the shape of EPSCs from Phf21b^Δ4/Δ4^ and Phf21b^+/+^ mice (Supplemental Figure 3, A and B). Moreover, induction of long-term potentiation (LTP) at the Schaffer collateral pathway was greatly impaired in Phf21b^Δ4/Δ4^ CA1 hippocampal neurons (Figure 4J).

**Figure 5:**
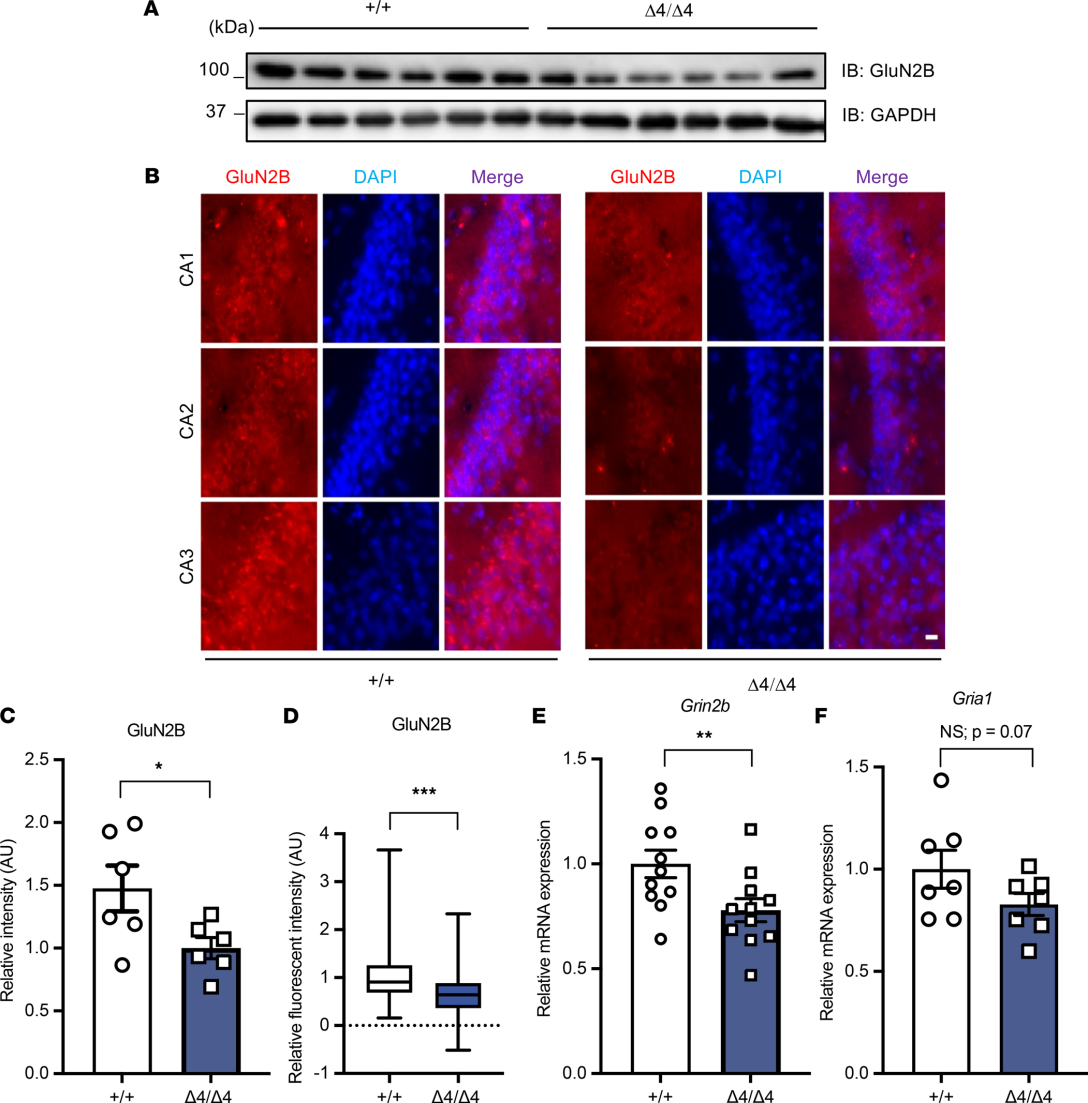
Decreased hippocampal GluN2B and Grin2b expression in Phf21b^Δ4/Δ4^ (Δ4/Δ4) mice. Representative images of (**A**) GluN2B immunoblot (IB) and (**B**) immunofluorescence staining of +/+ and Δ4/Δ4 hippocampi; scale bar: 20 μm. (**C**) Quantification of IB (*n* = 6/group) and (**D**) immunofluorescence (*n* = 3/group). (**E** and **F**) qRT-PCR of *Grin2b* and *Gria1* (*n* = 8–11/group). Values are presented as mean ± SEM (**C**, **E**, and **F**) or minimum to maximum and line at the median (**D**); Student’s *t* test/Mann-Whitney test; * *P* < 0.05; ** *P* < 0.01; *** *P* < 0.001.

The NMDAR (especially its subunits) is the classical mediator of activity-dependent synaptic plasticity in hippocampal CA1 region. Therefore, we examined the hippocampal levels of GluN2B, a pivotal subunit of NMDAR that mediates synaptic plasticity and is involved in neural development, using immunoblotting and immunofluorescence staining in Phf21b^Δ4/Δ4^ and Phf21b^+/+^ mice (Figure 5, A and B). We found that Phf21b^Δ4/Δ4^ mice had decreased GluN2B levels (Figure 5, C and D). Furthermore, immunofluorescence staining showed that PHF21B depletion impaired hippocampal GluN2B cluster intensity in CA1, CA2, and CA3, which further indicates GluN2B function (Figure 5, B and D). Additionally, expression of the gene encoding GluN2B (*Grin2b*) was decreased in Phf21b^Δ4/Δ4^ mice (Figure 5E). However, the mRNA level for the gene encoding GLUR1 (*Gria1*) tended to decrease but was not significantly changed (*P* = 0.07; Figure 5F). Collectively, these data suggest that decreased PHF21B expression leads to hippocampal synaptic dysfunction.

### PHF21B modulates the expression of genes involved in neurotransmission.

Earlier studies have described PHF21B as being involved in regulating gene expression. To gain mechanistic insight into the role that PHF21B plays in mediating neurotransmission, we performed genome-wide transcriptome profiling by using hippocampal tissues from the Phf21b^+/+^ and Phf21b^Δ4/Δ4^ animals.

**Figure 6:**
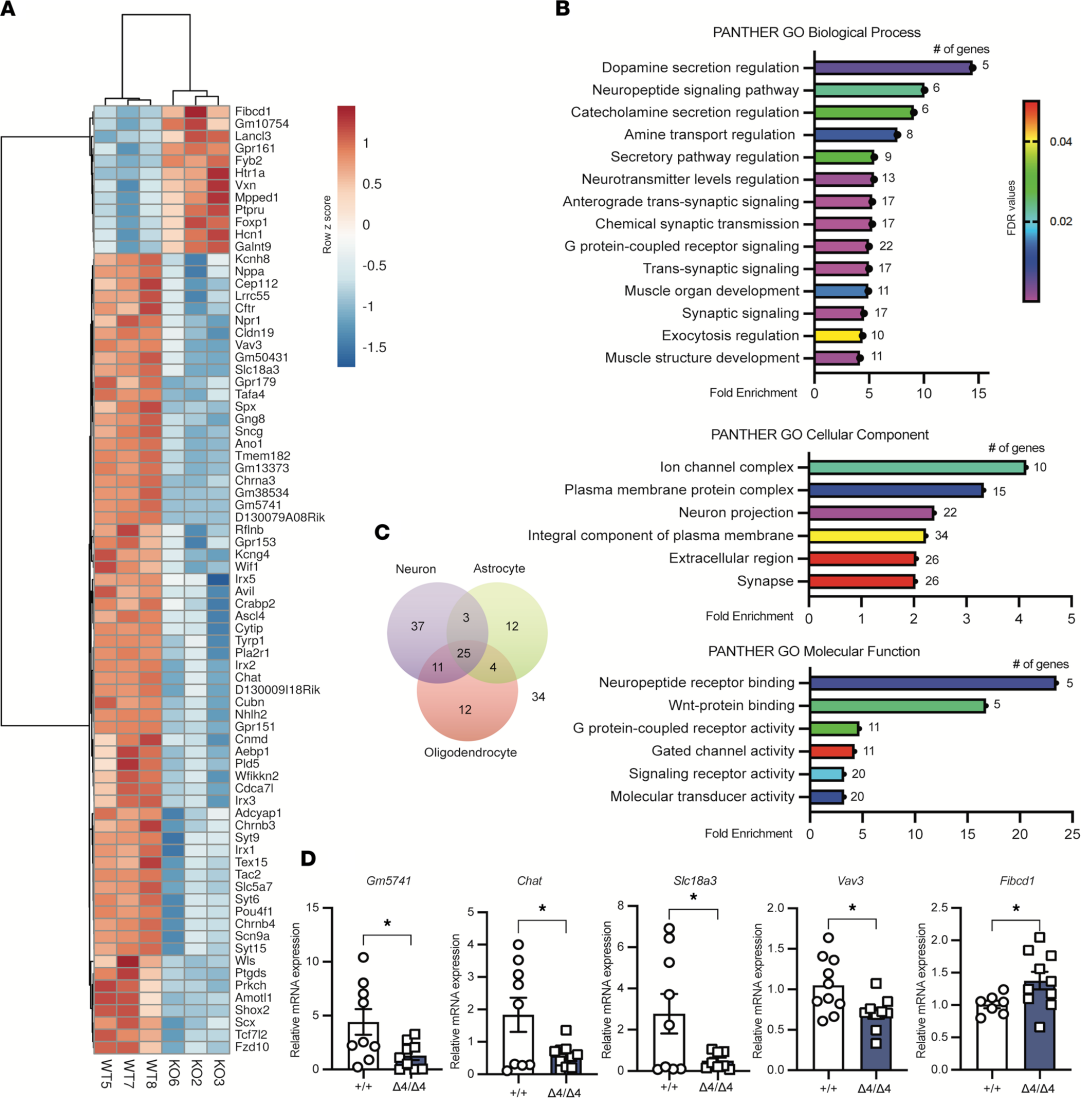
PHF21B modulates the expression of genes involved in neurotransmission. (**A**) Heatmap of differential gene expression (–2 ≤ fold change ≥ 2) of Phf21b^+/+^ (+/+) and Phf21b^Δ4/Δ4^ (Δ4/Δ4) hippocampal tissues; *n* = 3/group. (**B**) Top PANTHER gene ontology (GO) classifications associated with the differentially expressed genes (DEGs with FDR < 0.05 and gene count > 5) in +/+ and Δ4/Δ4 hippocampal tissues. (**C**) Venn diagram depicting the number of DEGs enriched in neurons, astrocytes, and oligodendrocytes. (**D**) qRT-PCR validation of DEGs (from left to right: *Gm5741*, *Chat*, *Slc18a3*, *Vav3*, and *Fibcd1*) with significant fold changes between +/+ and Δ4/Δ4 hippocampi were performed once; *n* = 10/group. Values are presented as mean ± SEM; Student’s or Welch’s *t* test; * *P* < 0.05.

We found a set of differentially expressed genes (*n* = 139) in the tissues with an FDR of less than 0.1 (Figure 6A and Supplemental Table 3; RNA-Seq data have been deposited under the accession number GSE201477, National Center for Biotechnology Information Gene Expression Omnibus [NCBI GEO] repository). The differentially expressed genes (DEGs) were enriched for synaptic processes, such as synaptic signaling, gated channel activity, and neurotransmitter levels (Figure 6B). The DEGs also showed enrichment for chemical synaptic transmission, which encompasses spontaneous and evoked release of neurotransmitters and all parts of synaptic vesicle exocytosis and cell-cell signaling (Figure 6B). Genes involved in neuropeptide signaling and G protein–coupled receptor signaling were also differentially expressed between the Phf21b^+/+^ and Phf21b^Δ4/Δ4^ hippocampal tissues (Figure 6B). A large majority of the DEGs were neuron specific (Figure 6C), further illustrating that PHF21B plays a role in modulating the expression of genes involved in synaptic plasticity. We were able to verify 5 of the 8 randomly selected DEGs (63%) with significant fold changes by using quantitative reverse transcriptase polymerase chain reaction (qRT-PCR). Specifically, we were able to verify the expression changes for the *Gm5741*, *Chat*, *Slc18a3*, *Vav3*, and *Fibcd1* genes (Figure 6D) but were not able to validate *Nppa*, *Gm38534*, and *D130009I18Rik*.

### PHF21B regulates transcription through its interaction with H3K9ac, H3K9me2, and CREB, and it interacts with H3K36me3.

**Figure 7:**
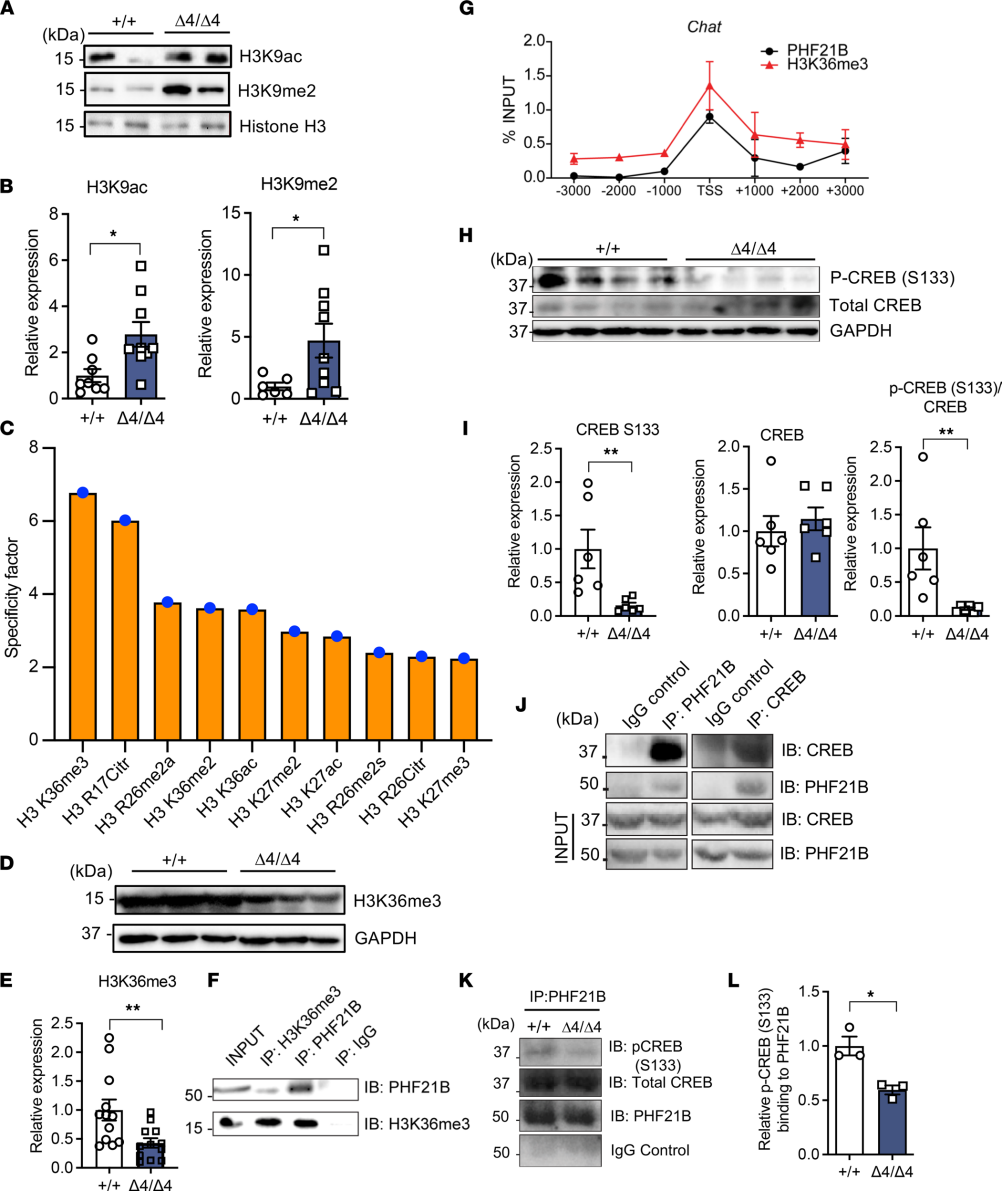
PHF21B regulates transcription through its interaction with H3K9ac, H3K9me2, and CREB and interacts with H3K36m3. (**A**) Representative Western blot images of H3K9ac and H3K9me2 expression levels in Phf21b^+/+^ (+/+) and Phf21b^Δ4/Δ4^ (Δ4/Δ4) hippocampal tissues. Histone H3 serves as the loading control; each lane is an individual animal; and (**B**) quantification; *n* = 6–9/group. (**C**) Graphical representation of purified recombinant PHF21B interaction specificity with several modified histone peptides. (**D**) Representative Western blot images of hippocampal H3K36me3 expression levels and (**E**) quantification; *n* = 6–12/group. (**F**) Representative immunoblot images of H3K36me3- and PHF21B-immunoprecipitated fractions from WT hippocampal tissues; *n* = 3. (**G**) Chromatin immunoprecipitation of genomic regions around the transcriptional start site (TSS) of the mouse *Chat* gene using anti-PHF21B or anti-H3K36me3 antibodies; “-” denotes base pairs upstream of the TSS; “+” denotes base pairs downstream from the TSS. (**H**) Representative immunoblot images of p-CREB (S133) and total CREB expression levels in +/+ and Δ4/Δ4 hippocampal tissues; GAPDH serves as the loading control; each lane is an individual animal; and (**I**) quantification; *n* = 6/group. (**J**) Immunoblots of PHF21B- and CREB-immunoprecipitated fractions using WT hippocampal tissues; *n* = 3. (**K**) Co-immunoprecipitation of PHF21B and p-CREB (S133) using +/+ and Δ4/Δ4 hippocampal tissues and (**L**) relative binding of PHF21B to p-CREB (S133) in +/+ and Δ4/Δ4 hippocampal tissues; *n* = 3. Values are presented as mean ± SEM; Student’s *t* test/Mann-Whitney test; * *P* < 0.05; ** *P* < 0.01.

Previous studies have reported an epigenetic role for PHF21B in transcriptional repression. Therefore, we examined the epigenetic landscape changes in the Phf21b^Δ4/Δ4^ hippocampus to validate this possibility. We found that markers of transcription activation (H3K9ac) and transcription repression (H3K9me2) (Figure 7, A and B) were increased in Phf21b^Δ4/Δ4^ hippocampal tissues as compared with Phf21b^+/+^ tissues, which suggests that PHF21B could have roles in both transcriptional activation and repression. These results are in line with Basu et al.’s findings (18). Moreover, these histone modifications bind to the *Grin2b* gene promoter region (22, 23). The relatively greater increase in the repressive H3K9me2 (~6 folds) than the activating H3K9ac (~3 folds) coupled with the higher specificity of PHF21B to H3K9me2 than H3K9ac (18) contributed to the net elevation of H3K9me2 activity. Thus, decreasing *Grin2b* gene and GluN2B (Figure 5, C and E) expression in Phf21b^Δ4/Δ4^ hippocampi decreased glutamatergic neurotransmission in CA1.

We examined PHF21B’s chromatin binding because the PHF21B protein contains a PHD domain that binds to DNA. We used purified PHF21B protein in a MODified Histone Peptide Array (Active Motif) to test its affinity for specific histone modifications. The array revealed preferential binding of PHF21B to H3K36me3, a histone modification marker associated with transcriptionally active genes (Figure 7C and Supplemental Figure 4). Furthermore, immunohistochemistry studies showed that WT hippocampal neurons had a greater colocalization index of PHF21B with H3K36me3 compared with either protein with DAPI (an established marker of transcriptionally silent heterochromatin) (Supplemental Figure 5, A–C). Furthermore, PHF21B and H3K36me3 protein levels were reduced in the hippocampus of Phf21b^Δ4/Δ4^ animals (Figure 1C and Figure 7, D and E). Notably, we further verified this interaction via co-immunoprecipitation and detected PHF21B in the H3K36me3-immunoprecipitated fraction (Figure 7F). Additionally, both PHF21B and H3K36me3 were found to be present at the promoter region, specifically at the transcriptional start site, of the PHF21B target gene *Chat* (Figure 7G). To further establish PHF21B as a regulator of transcriptional activation, we measured the levels of the well-known transcription factor CREB (24, 25) and its phosphorylated active form, p-CREB — phosphorylated at the serine residue 133 (S133) — and found that the latter was decreased in Phf21b^Δ4/Δ4^ hippocampi (Figure 7, H and I). We then performed co-immunoprecipitation of PHF21B with CREB and p-CREB (S133). Our results showed that PHF21B interacts with CREB (Figure 7J). Also, PHF21B interaction with p-CREB (S133) may be decreased in Phf21b^Δ4/Δ4^ hippocampal tissues (Figure 7, K and L); however, this finding may be confounded by the reduced p-CREB (S133) levels (to ~25%; Figure 7I) and PHF21B levels (to ~60%; Figure 1C) in Phf21b^Δ4/Δ4^ hippocampi compared with WT. Together, these data uncovered what we believe is a previously unknown functional role of PHF21B as a mediator of transcriptional activation through CREB and p-CREB (S133) and established H3K36me3 as a potentially novel interactor for PHF21B. PHF21B also mediates transcription activation and repression by binding to H3K9ac and H3K9me2.

## Discussion

Here we elucidated a mechanistic role for PHF21B in regulating synaptic plasticity via controlled transcriptional activation. PHF21B depletion led to decreased neurotransmission and induction of LTP, which ultimately manifested as social memory deficits in a Phf21b^Δ4/Δ4^ mouse model. We have presented structural and functional evidence of what we believe is a previously unrecognized functional role for PHF21B in transcriptional activation.

In previous work, we were the first to show that this PHF protein was expressed and had a function in the brain (19). Basu et al. studied PHF21B during embryogenesis based on our original observation. PHF21B is induced during neurogenesis and exhibits a distinct spatiotemporal expression pattern during cortical development (18). Depletion of PHF21B in vivo inhibited neuronal differentiation. It is noteworthy that PHF proteins regulate plant roots and mammalian cerebral cortex development. We show that PHF21B functions go well beyond development and are critical for normal brain functioning that affects social behavior.

Previous studies postulated that PHF21B functions similarly to PHF21A as a transcriptional repressor due to their sequence homology (26). Therefore, PHF21B had been associated with epigenetic repressors like histone deacetylase HDAC2 and KDM1A (18). However, in our data set, PHF21B was associated with a noticeably more prominent number of downregulated genes with more significant fold change (~3 orders of magnitude) than upregulated ones. Consequently, PHF21B potentially functions instead of or also as an enabler of transcriptional activation since PHF21B binds to the histone modification H3K9ac (Figure 7, A and B) and CREB, which may contribute to the phenotype of PHF21B depletion. The recruitment of CREB may also mediate transcription activation by PHF21B binding to the histone modification H3K36me3 at the transcriptional start sites of target genes. However, further studies are needed to establish whether H3K36me3 is necessary for transcription regulation. Both H3K9ac and H3K9me2 bind to the *Grin2b* gene promoter region (22, 23) and have opposite roles in regulating *Grin2b* gene expression. The repressive H3K9me2 decreased NR2B expression and glutamatergic neurotransmission in the Phf21b^Δ4/Δ4^ mice.

In another line of investigation, a similar role in recruitment was also reported in PHF21A because it is involved in neuron-specific gene repression by serving as a scaffold protein in the BRAF-HDAC complex (27). Although PHD fingers are more commonly reported to recognize methylation of H3K4 (28), binding to methylated H3K36 has also been documented, at least in yeast (29). Further, most of the DEGs (98 out of the 139, 70.5%) identified in our RNA-Seq of Phf21b^Δ4/Δ4^ versus Phf21b^+/+^ hippocampal tissues are predicted to possess CREB binding sites in their promoter/enhancer regions (30). Thus, in combination with previous reports, the data set presented here describes a dual function for PHF21B in transcriptional regulation, presumably governed by the type of histone modification it binds to and the subsequent recruitment of cofactors.

A vast body of evidence links complex neurobehavioral disorders to epigenetic gene regulatory mechanism aberrations (31–34). However, a major challenge in uncovering these mechanisms is the unequivocal identification of the molecules involved and their connections to genes. Although the Phf21b^Δ4/Δ4^ mouse generated here is a knockdown model, marked gene changes were still observed by RNA-Seq, with a large proportion of the DEGs being related to synaptic processes (e.g., synaptic and trans-synaptic signaling, chemical synaptic transmission, regulation of neurotransmitter). This finding underscores the sensitivity of the gene expression mechanisms controlling these genes to changes in the levels of their epigenetic regulators, which is the PHF21B in this case. Moreover, expression changes of genes involved in glutamate binding, activation of AMPAR, synaptic plasticity, AMPAR trafficking (such as *Prkcb*, *Ap2b1*, *Akap5*), and negative regulation of NMDAR-mediated neuronal transmission (such as *Camk4* and *Ppm1e*) may also have contributed to deficits in neurotransmission and in the inability of Phf21b^Δ4/Δ4^ CA1 neurons to induce LTP. This functional deficit likely manifested as the impaired social memory observed in the Phf21b^Δ4/Δ4^ mice. The discovery that PHF21B is a common regulator of a sizable group of synaptic genes is meaningful to expanding the knowledge of epigenetic mechanisms of synaptic plasticity and behavior.

Dysfunction of PHD-containing proteins in the brain is often associated with neurobehavioral disorders. Intellectual disability and epilepsy in human patients were found to be caused by de novo truncating variants in PHF21A (35, 36). Moreover, increased seizure sensitivity, emotional defects, and cognitive impairment were reported in PHF24-null rats (37). In contrast, loss of PHF8 conferred resistance to depressive- and anxiety-like behaviors in mice (38). We report here impaired social memory as a result of PHF21B depletion, further highlighting the importance of PHD proteins to proper brain function.

Social memory is vital for many social behaviors (39). Aberrant social behaviors manifest in neuropsychiatric disorders, such as autism, major depression disorder, and schizophrenia (40). The hippocampus is essential for encoding social memory (41–43). Of note, the Phf21b^Δ4/Δ4^ mice displayed normal social recognition but impaired social memory, as they did not habituate to the familiar stranger. Decreased GluN2B levels, impaired LTP, reduced synaptic efficacy, and altered expression of synaptic genes in the hippocampi of Phf21b^Δ4/Δ4^ mice likely contributed to their social memory deficits, as CA1 plays a specific role in social memory storage (43).

Finally, phosphorylation of CREB at S133 has been shown to be important for late-phase LTP (41, 44, 45) and consolidation of social memory (46–48); p-CREB (S133) expression levels were reduced in Phf21b^Δ4/Δ4^ hippocampi. Acetylcholine biosynthesis or neuropeptide signaling may have contributed to social memory deficits in the Phf21b^Δ4/Δ4^ mice. *Chat*, which encodes the enzyme that catalyzes the biosynthesis of acetylcholine, was one of the top DEGs in our RNA-Seq. It was decreased in Phf21b^Δ4/Δ4^ hippocampal tissues. It is noteworthy that administering the acetylcholine antagonist scopolamine blocks social memory formation (49). Dysregulated neuropeptide signaling may lead to social memory deficits. For example, loss of function of the oxytocin gene (50), or its receptors (51), has been reported to lead to social amnesia in mice. PHF21B is highly expressed in the pituitary gland (52), an established CNS hub of neuropeptide signaling. Therefore, in future studies, it would be intriguing to investigate the role of PHF21B in modulating neuropeptide activity and the effects of both PHF21B and neuropeptide activity on downstream behaviors such as social memory.

In summary, we have shown that PHF21B functions as a key upstream regulator of gene expression events linked to neurotransmission regulating social memory. Upcoming work will focus on CA2 and the prefrontal cortex as these regions have specific roles in social memory (43, 53), and Phf21b^Δ4/Δ4^ mice have decreased GluN2B clustering in CA2 (Figure 4F) and morphological defects in the cortex. In addition, future studies are needed to establish PHF21B as a candidate therapeutic target for the underlying synaptic dysfunction that is widely prevalent in neurobehavioral disorders.

There are limitations of this study, including the fact that PHF21B is highly expressed in neural tissues, comprising peripheral and gastrointestinal tract nerves that can affect the production of neurotransmitters, which may be a confounder in our studies, where we used a mouse model with whole-body PHF21B depletion.

## Methods

### Animals.

Phf21b^Δ4/Δ4^ animals were generated by using standard CRISPR/Cas9 methodology at the South Australia Genome Editing Facility of the University of Adelaide. Two CRISPR guides were designed to disrupt exon 4 of the Phf21b gene in mice. The guide sequences used were: 5′ CRISPR guide: CGGTCCCTGCCCGGGGTGAC; and 3′ CRISPR guide: CTGCACTTTGATGCCGTCGC. Founders were screened by PCR and confirmed by Sanger DNA sequencing. The animals were maintained on a C57BL/6J genetic background. Mice were genotyped by PCR by using genomic DNA from tail clip samples. Mice were group-housed in humidity- and temperature-controlled (22 ± 1°C) rooms with a 12-hour light/12-hour dark cycle. Animals were given ad libitum access to food and drinking water. Young adult male and female Phf21b^+/+^ and Phf21b^Δ4/Δ4^ littermates between 2 and 6 months old were used for all experiments, unless otherwise specified.

### Behavioral testing.

All behavioral tests were conducted in dedicated experiment rooms during the lights-on cycle. Equal numbers of adult (8–12 weeks old) male and female animals were used. Preliminary testing showed no differences in the results obtained from both sexes, and hence the data obtained are from both sexes. The mice were put into the testing rooms about an hour before testing. All test sessions were video-recorded and analyzed by using either ANY-maze 2.0 (Stoelting Co.) or EthoVision XT 11.5 (Noldus Information Technology) software. All behavioral procedures were performed with an experimenter blinded to the genotypes of the mice. Detailed description of behavioral protocols can be found in the Supplemental Methods.

### Electrophysiology.

Male and female Phf21b^+/+^ and Phf21b^Δ4/Δ4^ mice (2–3 months old) were used. The final LTP data shown were collected from male mice, and final input/output and paired-pulse ratio data shown were collected from female mice.

After euthanasia by decapitation, mouse brains were quickly moved into a semifrozen sucrose dissection solution consisting of 75 mM sucrose, 87 mM NaCl, 2.5 mM KCl, 1.25 mM NaH_2_PO_4_, 7 mM MgCl_2_, 25 mM NaHCO_3_, 10 mM glucose, 0.5 mM CaCl_2_, 1.3 mM ascorbic acid (54), and oxygenated by bubbling with an admixture of 95% O_2_ and 5% CO_2_. Transverse hippocampal slices (350 μm) were obtained by using a Leica VT1200 S (Leica Biosystems). Slices were transferred in an artificial cerebrospinal fluid (aCSF) solution consisting of 126 mM NaCl, 18 mM NaHCO_3_, 2.5 mM KCl, 2.4 mM MgCl_2_, 1.2 mM CaCl_2_, 1.2 mM NaH_2_PO_4_, and 11 mM glucose (55) and oxygenated with 95% O_2_ and 5% CO_2_. Slices were first incubated at 32°C for 45 minutes, then kept at room temperature (RT) for the remainder of the experiment.

Standard whole-cell patch clamp was performed on visually identified CA1 pyramidal neurons. Slices were transferred to a recording chamber and perfused with oxygenated aCSF (containing 100 μM picrotoxin) at 32°C. Patch pipette internal solution consisted of 128 mM potassium gluconate, 8 mM NaCl, 0.4 mM EGTA, 2 mM Mg-ATP, 0.3 mM Na-GTP, and 5 mM QX-314, buffered with 10 HEPES [pH 7.3]. Electrode resistance was 6–8 MOhm. Neurons were voltage-clamped at –60 mV. Presynaptic stimulation of Schaffer collateral afferents in stratum radiatum was delivered at 0.033 Hz with a bipolar metal electrode (FHC Inc.) to evoke EPSCs. After 10 minutes of baseline recording stable EPSCs, the cell was voltage-clamped to +30 mV, and a train of 240 stimuli at 4 Hz was applied to induce LTP. After induction, the cell was clamped to –60 mV, and EPSCs were recorded for 50 minutes. LTP was quantified by the ratio of average amplitudes of EPSCs after induction over the baseline average. For input/output relationship, EPSCs were evoked by a series of stimuli with increasing intensity (from 20 to 2400 μA). The amplitude of each EPSC (i.e., output) was measured and plotted against the corresponding stimulation intensity (i.e., input). Data were collected by using a MultiClamp 700B amplifier (Axon Instruments) and pCLAMP 11 software (Molecular Devices).

### Transcriptome profiling.

RNA-Seq was performed by the Molecular Analysis Core Facility at State University of New York Upstate Medical University. RNA was extracted from Phf21b^+/+^ and Phf21b^Δ4/Δ4^ mouse hippocampal issues by using an RNeasy Mini Kit (Qiagen). RNA quality and quantity were assessed by using an RNA 6000 Nano Kit (Agilent Technologies) on an Agilent 2100 Bioanalyzer system (Agilent Technologies). For library prep, 500 ng total RNA was used as input to the TruSeq Total RNA Library Prep Ribo-Zero Gold Kit (Illumina). Library size distribution was determined by using a DNA 1000 Kit (Agilent Technologies) on the Agilent Bioanalyzer, and libraries were quantified by using a Qubit double-stranded DNA HS Assay Kit (Thermo Fisher Scientific) on a Qubit 3.0 fluorometer (Thermo Fisher Scientific). Libraries were sequenced with single-end 75 bp on a NextSeq 500 (Illumina) instrument. An average 32 million single-end 75 bp reads per sample were generated from sequencing. Sequencing quality was assessed by FastQC (v0.11.8) (56). Low-quality bases/reads and adaptors were removed from reads by Trimmomatic (v0.39) (57). The trimmed reads were mapped to GENCODE GRCm38 release M24 mouse reference genome by using STAR aligner (v2.7.3a) (58). Reads mapped to genes were summarized by the featureCounts program (59) in subread v1.6.4. Genes were filtered by counts per million ≥ 1 in at least 2 samples, and data were normalized to effective library size by edgeR (v3.28.1) (60). Differential gene expression analyses were performed by using edgeR. RNA-Seq data were deposited under the accession number GSE201477, NCBI GEO repository. Threshold for FDR was set at less than 0.1, and fold changes greater than ±1.3 were considered statistically significant. RNA-Seq and qRT-PCR confirmation studies were performed once. GO term enrichment analysis was performed by using the PANTHER database (61, 62), with the enrichment criteria of *P* < 0.05 and gene count > 5. Heatmap for visualization of differential gene expression was generated by using ClustVis (63).

### RNA extraction, cDNA conversion, and qRT-PCR.

Brain tissues were homogenized on ice by using a Dounce homogenizer. Total RNA was extracted with TRIzol (Thermo Fisher Scientific). Extracted RNA was resuspended in nuclease-free water and stored at –80°C until use. cDNA was synthesized by using oligo(dT) primers and iScript cDNA Synthesis Kit (Bio-Rad Laboratories, Inc.). qRT-PCR was performed by using SYBR Select Master Mix for CFX (Thermo Fisher Scientific) on a CFX384 Touch Real-Time PCR Detection System (Bio-Rad Laboratories, Inc.). Gene expression values were normalized to that of the housekeeping gene *Gapdh*. Primer sequences are listed in Supplemental Table 4.

### Protein extraction and immunoblotting.

Brain tissues were homogenized on ice in RIPA cell lysis buffer (10 mM Tris-HCl [pH 8.0], 140 mM NaCl, 1 mM EDTA, 1 mM PMSF, 1% Triton X-100, 0.1% sodium deoxycholate, 0.1% SDS) by using a Dounce homogenizer. For immunoprecipitation/co-immunoprecipitation, tissue samples were homogenized in RIPA lysis buffer without SDS; approximately 1 mg clarified total protein lysis was rotationally incubated with protein A/G agarose beads and indicated antibody or IgG control (4 μg) at 4°C overnight. Precipitated proteins were incubated and eluted with 50 μL 0.1 M glycine at pH 2.5 to 3 at RT for 10 minutes after wash of immunoprecipitation beads, and then 1 μL 10 N NaOH_2_ was immediately added to elution sample to neutralize pH. Protein samples were denatured and separated on SDS-PAGE gels under reducing conditions. After transferring onto PVDF membranes, they were blocked with 5% BSA in Tris-buffered saline (TBS) containing 0.1% Tween 20. The membranes were incubated overnight at 4°C with primary antibodies. Primary antibodies used were anti-CREB (1:500, Cell Signaling Technology, 9197), anti-GAPDH (1:10,000, Cell Signaling Technology, 5174), anti-H3K9ac (1:500, MilliporeSigma, 06-942-S), anti-H3K9me2 (1:500, Abcam, 1220), anti-H3K36me3 (1:500, Thermo Fisher Scientific, Ma5-24687), anti-Histone H3 (1:500, MilliporeSigma, 06-755), anti–p-CREB (S133) (1:500, Cell Signaling Technology, 9198), anti-PHF21B (1:2000, Invitrogen, PAS-7686), and anti-GluN2B (1:500, ProteinTech, 21920-1-AP). Amersham ECL horseradish peroxidase–linked secondary antibodies (1:5000, Cytiva, NA934V and NA931V) were used to detect the primary antibodies. Blots were developed by using SuperSignal Chemiluminescent Substrate (Thermo Fisher Scientific) and imaged with a ChemiDoc MP Imaging System (Bio-Rad Laboratories, Inc.). Band densitometry analysis was done on the ImageJ software (NIH).

### Golgi staining.

Golgi staining was performed by using an FD Rapid GolgiStain Kit (FD NeuroTechnologies Inc.) with minor modifications as described (64). Sectioning was performed on a cryostat maintained at –20 to –22°C. Sections of 150 μm were obtained and mounted onto gelatin-coated glass microscope slides. Air-dried sections were washed with double-distilled water and immersed in the staining solution for 10 minutes, followed by graded dehydration in ethanol. Xylene was used to clear the sections, which were then mounted with Permount (VWR International, LLC.).

### Sample preparation, immunohistochemistry, and image acquisition and analysis.

For obtaining brain sections, deeply anesthetized mice, with ketamine (150 mg/kg)/xylazine (10 mg/kg), were perfused through the heart with ice-cold 0.1 M phosphate buffer, followed by ice-cold 4% paraformaldehyde (PFA). Harvested brains were postfixed for 24 hours and then transferred to 30% sucrose solution for cryoprotection; 20 or 40 μm thick sections were obtained for immunostaining. For obtaining primary neuronal cultures, primary neurons were dissociated from postnatal day 0 pups from Phf21b^+/+^ and Phf21b^Δ4/Δ4^ animals as described (65). Dissociated neurons were plated onto glass coverslips and maintained in a humidified incubator at 37°C and 5% CO_2_ until use. At day in vitro 9, the neurons were washed with PBS and fixed with 4% PFA-containing PBS.

For immunostaining, samples were blocked with 5% donkey serum in TBS containing 0.1% Triton X-100 and incubated overnight with primary antibodies. Images were taken on a Zeiss LSM780 confocal microscope system. Laser intensity and gain parameters were kept constant for all images in the same experiment. Signal intensities were quantified by using ImageJ software. Neurite tracings were made by using the Simple Neurite Tracer ImageJ plug-in (66). Analysis of dendritic arborization was performed by using the Sholl plug-in in ImageJ (67). Imaging and analysis of Golgi-stained brain sections were done for secondary branches. Fluorescence signal colocalization was quantified using the Colocalization Colormap plug-in in ImageJ (68). The primary antibodies used were anti–cleaved caspase-3 (1:500, Cell Signaling Technology, 9664), anti-Doublecortin–Alexa Fluor 488 (1:500, Santa Cruz Biotechnology, sc-271390) anti-GFAP (1:500, MilliporeSigma, mab360), anti-GLUR1 (1:500, MilliporeSigma, abn241), anti-GluN2B (1:500, ProteinTech, 21920-1-AP), anti-MAP2 (1:1000, MilliporeSigma, m9942), anti-NEUN (1:500, MilliporeSigma, mab377), anti-H3K36me3 (1:500, Thermo Fisher Scientific, Ma5-24687), anti-PHF21B (1:500, Invitrogen, PAS-7686), and anti–PSD-95 (1:500, Abcam, 12093). Alexa Fluor secondary antibodies (1:500, Thermo Fisher Scientific, A21432, A11055, A31571, and A31572) were used. Nuclei were stained with DAPI.

### Histone peptide array.

We obtained a human *PHF21B* Myc-tagged ORF clone (Origine), transfected it into HEK293 cells (ATCC), and produced and purified recombinant human PHF21B protein to incubate it with the MODified Histone Peptide Array (Active Motif) following the manufacturer’s protocol. A charge-coupled device camera was used to capture images, and image analyses were performed using the freely available Active Motif’s Array Analyze Software. This experiment was performed once.

### Data and materials availability.

All unique/stable reagents and animals generated in this study are available from the corresponding author with a completed material transfer agreement. A list of all DEGs in our RNA-Seq data is provided in Supplemental Table 3. Further information and requests for resources and reagents should be directed to, and will be fulfilled by, the corresponding author, Ma-Li Wong.

### Statistics.

Experiments were repeated at least 2 times, unless otherwise noted. A total of 4–6 image fields from each brain section were analyzed, with 3–5 sections immunostained per brain. A minimum of 30 cells were analyzed for each condition for primary neuron cultures. Statistical testing was performed by using GraphPad Prism. We used 2-tailed Student’s *t* test, 1-way ANOVA, 2-way ANOVA, or mixed effects analysis for normally distributed data and Welch’s *t* test, Wilcoxon-Mann-Whitney test, or Kruskal-Wallis test for non-normal data. A *P* value less than 0.05 was considered significant.

### Study approval.

All animal experiments were performed according to approved protocols by the IACUC of the State University of New York Upstate Medical University and the Animal Ethics Committees of the South Australian Health and Medical Research Institute, Flinders University, and the University of Adelaide. All procedures followed the *NIH Guide for the Care and Use of Laboratory Animals* (National Academies Press, 2011) and the Australian Code for the care and use of animals for scientific purposes (69).

## Author contributions

JL and MLW conceived of the study. MDL, MW, and MLW designed the methodology. EWMC, QM, HR, CC, and AS conducted the investigation. EWMC, CZ, QM, and CL completed formal raw data analyses, and EWMC, QM, and MLW curated the raw data. TLS, WA, JL, WDY, XYL, and JL provided insights or reagents. Visualization was completed by EWMC, MLW, QM, CC, and CZ. All authors analyzed the processed data. EWMC wrote the original draft, which EWMC, TLS, MB, WDY, XYL, WA, JL, and MLW reviewed and edited. EWMC and MLW supervised the project. Funding was acquired by JL, MB, MLW, and WA.

## Supplementary Material

Supplemental data

## Version 1 07/22/2022

Electronic publication
