# Supplementary material for: The epigenetic reader PHF21B modulates murine social memory and synaptic plasticity–related genes
Source: JCI Insight. 2022 Jul 22;7(14):e158081. doi: 10.1172/jci.insight.158081 (PMC9431697; doi:10.1172/jci.insight.158081)
Supplement: Supplemental data [file jciinsight-7-158081-s018.pdf]

Supplemental material

Methods

PCR-based off-target analysis of CRISPR-mediated editing in Phf21b$^{\Delta4/\Delta4}$ mice. We used a PCR-based approach to validate the precision of CRISPR-mediated gene editing in generating *Phf21b*$^{\Delta4/\Delta4}$ mice. We first searched for potential off-targets by the guide sequences in the genome of the animals by using Cas-OFFinder (1) (Fig. S1A). PCR primers were designed for the three highest ranked off-target sites for each guide sequence (Table S1). The PCR was performed by using genomic DNA samples from the *Phf21b*$^{\Delta4/\Delta4}$ mice. PCR products were separated via agarose gel electrophoresis (Fig. S1B) and extracted for sequence confirmation via Sanger DNA sequencing (Fig. S1C). The selected sites did not show evidence of off-target CRISPR editing in the *Phf21b*$^{\Delta4/\Delta4}$ mice.

Behavioral assays: Open field. In the open field test, mice were placed in a 40 x 40 x 40 cm arena and allowed to explore freely for 10 min. For analysis, the center of the arena is prescribed as the 20 x 20 cm area in the middle.

Behavioral assays: Elevated-plus maze. In the elevated-plus maze test, mice were allowed to freely explore the elevated-plus maze for 5 min, during which the amount of time that they spent in the open arms versus the closed arms was measured.

Behavioral assays: Rotarod assay. Mice were placed on an accelerating (from 4 to 40 rpm) rotarod (IITC Inc.). Latency to fall from the rotarod was measured and recorded. The animals were given four trials a day for four consecutive days. Inter-trial interval was at least 15 min, during which the animals were returned to their home cages to rest.

Behavioral assays: Sucrose preference test. Mice were allowed free choice to drink from two identical bottles containing either water or 1% sucrose solution. The amount of intake from each bottle was measured after 48 h. The sucrose preference index is calculated as the amount of sucrose intake divided by the total intake of water and sucrose.

Behavioral assays: Tail suspension test. The test was performed as described (2). Mice were suspended from their tails for a period of 6 min. The amount of time they were mobile was scored. Immobility time is calculated by subtracting the mobility time from the total testing time of 6 min.

Behavioral assays: Forced swim test. The test was performed as described (3). Mice were placed in a clear Plexiglas cylinder filled with water for a period of 6 min. The amount of time they were mobile for the last 4 min of the test session was scored. Immobility time is calculated by subtracting the mobility time from the total scored time of 4 min.

Behavioral assays: Y-maze. Mice were allowed to freely explore all three arms of the Y-maze for a period of 10 min. Percentage of spontaneous alterations was calculated as a measure of spatial working memory, which is given by the total number of spontaneous alternations divided by the total number of arm entries minus 2, multiplied by 100.

Behavioral assays: Novel object recognition test. To test long-term recognition memory, the mice were allowed to explore two identical objects for 10 min. 24 h after the initial training session, one of the objects was replaced with a novel one, and the mouse was allowed to explore the two objects for 10 min. The novel object recognition index is given by the amount of time that the animal spends exploring the novel object divided by the total amount of time it spends exploring both objects in the test session.

Behavioral assays: Morris water maze. To test long-term spatial reference memory, the protocol as described in (4) was used. Briefly, mice were first trained for five consecutive days to reach an underwater platform in the Morris water maze. Four trials were given on each training day, with an inter-trial interval of 15 s, during which the mice were left on the platform. The start location was randomized for each trial. Mice were guided to the platform if they failed to reach it within the 1-min trial limit. On the sixth day, a probe trial was conducted, where the underwater platform was removed, and the mice were scored on their latencies to reach the platform location from a novel start location.

Behavioral assays: Social preference and social novelty tests. The social preference and social novelty tests were performed as described (5). Mice were habituated to the three-chambered setup and allowed to freely explore for 10 min. For the social preference test, the subject mouse was allowed to interact with either a juvenile stranger mouse placed in one of the side chambers, or an inanimate object in the other side chamber for a test period of 10 min. The social preference index is given the amount of time that the subject mouse spends interacting with the stranger mouse, divided by the total amount of time it spends interacting with both the stranger mouse and the object in the test session. For the social novelty test, the inanimate object was replaced with a new juvenile stranger mouse, and the subject animal was allowed to interact with either the familiar stranger or the new stranger for a test period of 10 min. The social novelty index is given by the amount of time that the subject mouse spends interacting with the new stranger, divided by the total amount of time that the subject mouse spends interacting with both the familiar and new stranger mice in the test session. All stranger mice were sex-matched to the subject animal, and the placements of the object and the stranger mice were counter-balanced between subjects.

Behavioral assays: Five-trial social memory test. The five-trial social memory test was performed as described in (6, 7). The subject animal was allowed to freely interact with a juvenile stranger animal of the same sex for four 1-min trials, with an inter-trial interval of 10 min. The same stranger animal was used for the four trials. For the fifth trial, a new stranger mouse was introduced to the subject mouse for 1 min. The interaction time of the subject animal with the stranger mouse in each trial was recorded.

Behavioral assays: Olfactory habituation/dishabituation test. Mice were tested for their ability to habituate and dishabituate to different odors, as described (8). Cotton swabs soaked with different odorants (water, vanilla, and urine from a conspecific animal) were presented to each mouse for three 2-min trials, with an inter-trial interval of 1 min. The amount of time that the animal spent investigating the odorant was recorded.

A

Bulge type	crRNA	DNA	Chromosome	Position	Direction	Mismatches	Bulge size
X	GTCACCCCGGGCAGGGACCGNGG	GgCACCCCaGGCAGGGtCCGAGG	chr8	13159733	-	3	0
DNA	GTCA-CCCCGGGCAGGGACCGNGG	GTCAGCCCCGGGagGGGACCGCGG	chr5	138995145	+	2	1
X	GTCACCCCGGGCAGGGACCGNGG	GTCACaCaGGGCAGGGACCtGGG	chr10	93806055	-	3	0
X	GCGACGGCATCAAAGTGCAGNGG	GCtAaGGCATCAAAGTGCAGAGG	chr13	88823611	-	2	0
X	GCGACGGCATCAAAGTGCAGNGG	cCGAgGGCATCgAAGTGCAGAGG	chr7	80802005	-	3	0
X	GCGACGGCATCAAAGTGCAGNGG	GCcttGGCATCAAAGTGCAGTGG	chrX	162783883	+	3	0

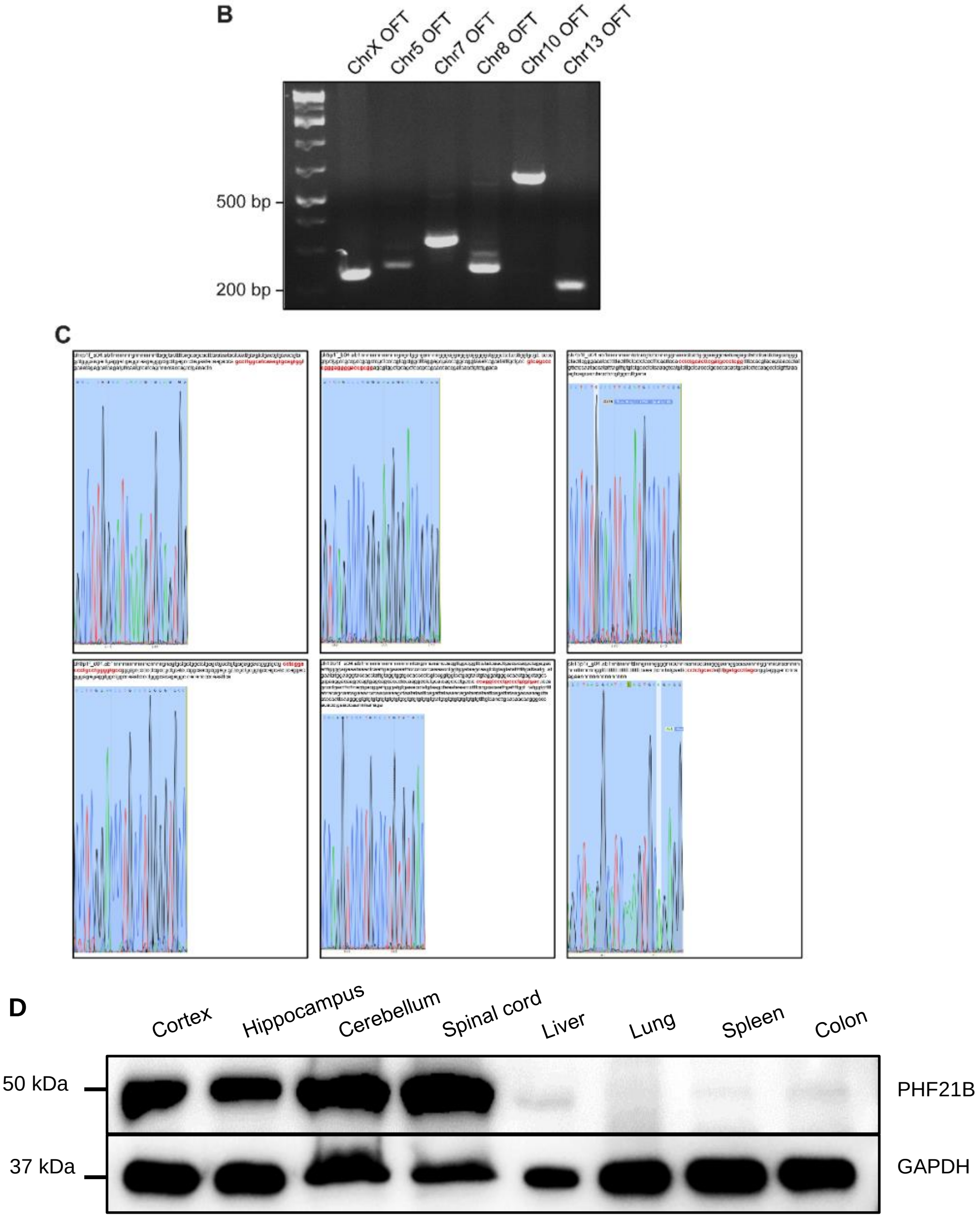

Figure S1. PCR-based off-target analysis of CRISPR-mediated editing in *Phf21b*$^{\Delta4/\Delta4}$ animals and verification of PHF21B expression. (**A**) Potential off-target sites identified via Cas-OFFinder. (**B**) Representative agarose gel image of PCR products from primers targeting potential off-target (OFT) sites in the genomic DNA of *Phf21b*$^{\Delta4/\Delta4}$ animals. (**C**) Sequence confirmation of intact off-target sites in the genomic DNA of *Phf21b*$^{\Delta4/\Delta4}$ mice. (**D**) Western blot of the wild-type mouse brain and peripheral tissues expressing PHF21B.

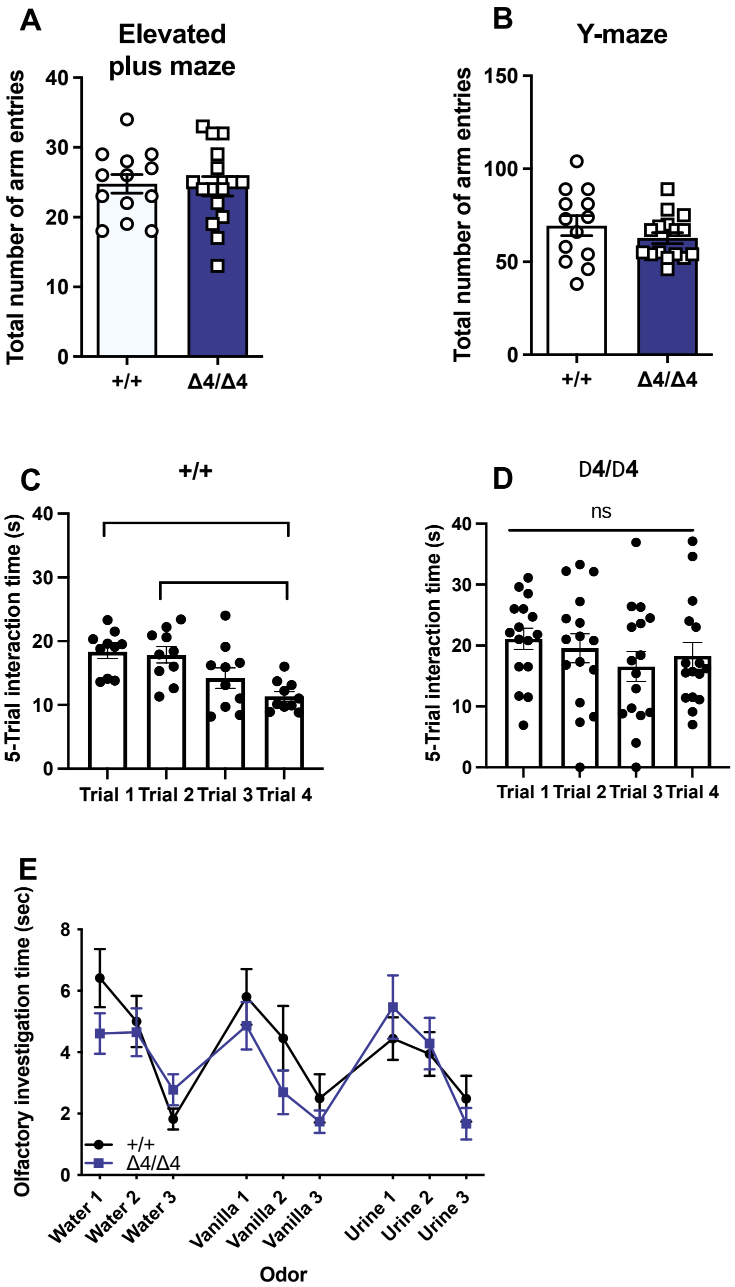

Figure S2. Behavioral characterization of *Phf21b*$^{+/+}$ (+/+) and *Phf21b*$^{\Delta4/\Delta4}$ (Δ4/Δ4) animals. (**A**) Total number of arm entries in the elevated plus maze test. (**B**) Total number of arm entries in the Y-maze test. (**C**) Interaction time in the 5-trial social test, one-way ANOVA with repeated measures in +/+ mice *P<0.05, **P<0.01 and (**D**) in Δ4/Δ4 mice. (**E**) Olfactory habituation/dishabituation results from +/+ and Δ4/Δ4 animals. n = 13-16. Values are presented as mean ± SEM.

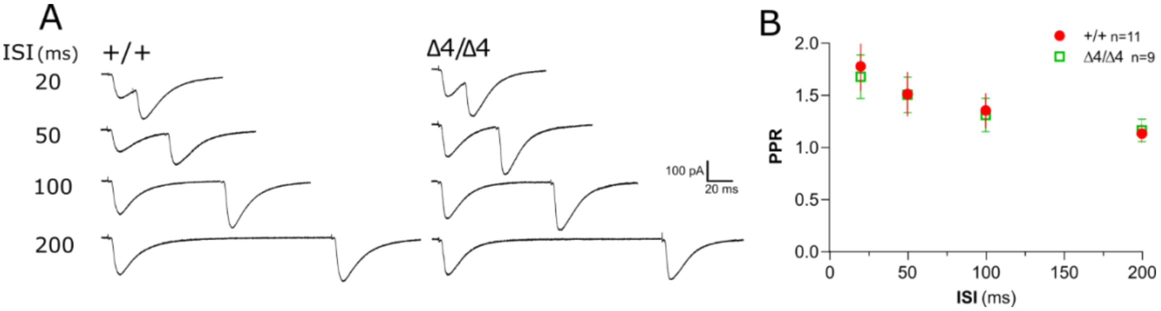

Figure S3. ***Phf21b*$^{\Delta4/\Delta4}$ (Δ4/Δ4) mice showed a normal pair-pulse ratio (PPR) in hippocampal CA1 synapses. A**. Representative traces of twin-EPSCs recorded from CA1 pyramidal neurons in *Phf21b* wild-type (+/+) and Δ4/Δ4 mice. ISI: inter-stimuli-intervals. **B**. Summary data averaged at same ISI across different neurons recorded from +/+ or Δ4/Δ4 mice. There is no difference between Δ4/Δ4 and +/+ mice, indicating that Δ4/Δ4 status does not alter the presynaptic release.

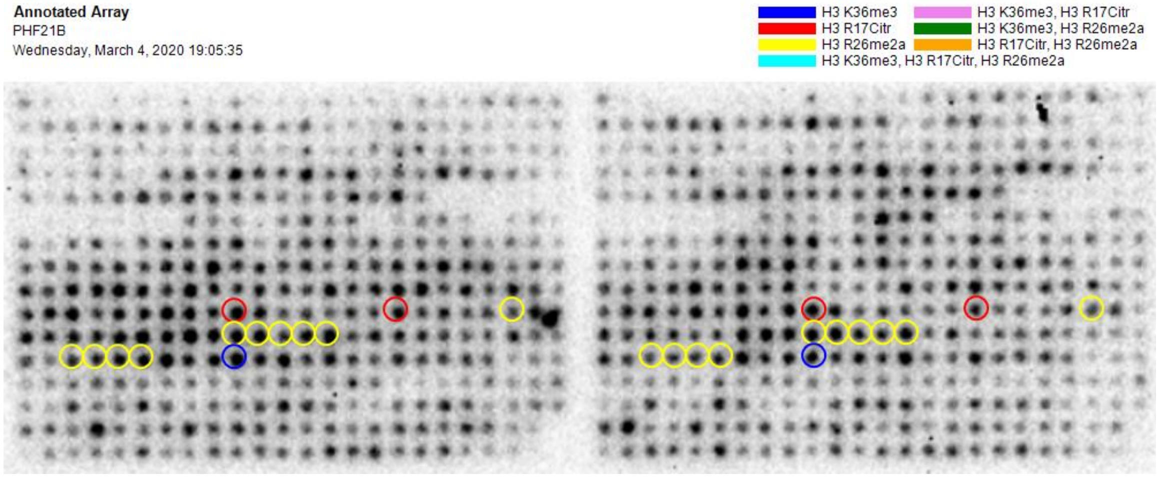

Figure S4. Enhanced chemiluminescence image of the MODiFied^{TM} Histone Peptide Array. This array was used to screen purified human recombinant PHF21B for its interactions with histone and their post-translational modifications. Single histone modifications were color coded.

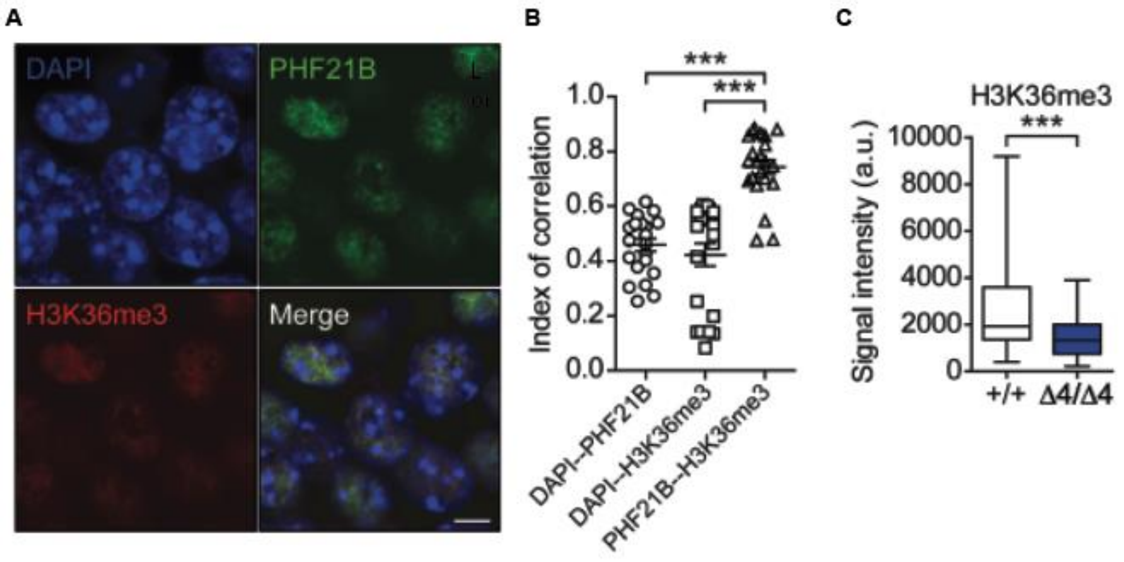

Figure S5. Colocalization of PHF21B with H3K36me3. (**A**) Representative images of PHF21B and H3K36me3 expression in the nuclei of wild-type CA1 neurons. (**B**) Index of correlation as a measure of co-localization of DAPI-, PHF21B-, and H3K36me3-positive immunofluorescence signals in the nuclei of wild-type CA1 neurons; n = 5; One-way ANOVA; Tukey's test *post hoc*. (**C**) Expression of H3K36me3 in +/+ and Δ4/Δ4 hippocampal tissues measured by its immunofluorescence signal intensity; n = 5; Student's *t*-test.

Table S1. Sequences of primers used for PCR-based off-target analysis of CRISPR-mediated *Phf21b* deletion.

Target	Forward sequence (5′ → 3′)	Reverse sequence (5′ → 3′)	Expected product size (bp)
ChrX	GCAGTGTGCTGATCCAGAGC	CAGACTGGTACAAGCTGATGAC	231
Chr5	ACAGAGTCGCTCCGGG	GTCCAGACAGGTGATCGGTG	244
Chr7	CAGGGCCTGTGGACTTTACC	CAAGGCCATGGGAAGGTAGG	320
Chr8	GTCTGAGCCGCTCTGGAAG	GAATTTGGGGGTGTGAGCCT	238
Chr10	CCAAGGACCATTCTGACATCT	CCTTTACTGGTTCAGGTGTGG	653
Chr13	ATGTTTTCGGAGTGGGTCGC	GAACAGAGGCGGGGATAGGA	201

Table S2. Posthoc Sidak's multiple comparison test of mixed ANOVA test of +/+ and Δ4/Δ4 mice

Fixed effects (type III)	P value	P value summary	F (DFn, DFd)
Intersections	<0.0001	****	F (78, 4368) = 143.0
Genotype	0.0662	ns	F (1, 56) = 3.510
Intersections x Genotype	<0.0001	****	F (78, 4368) = 1.744

Distance from soma (μm)	Predicted (LS) mean diff.	95.00% CI of diff.	Summary	Adjusted P Value
10	0.6952	-0.8174 to 2.208	ns	>0.9999
15	0.2548	-1.258 to 1.767	ns	>0.9999
20	0.04286	-1.470 to 1.555	ns	>0.9999
25	0.481	-1.032 to 1.994	ns	>0.9999
30	-0.03333	-1.546 to 1.479	ns	>0.9999
35	-0.3405	-1.853 to 1.172	ns	>0.9999
40	-1.017	-2.529 to 0.4959	ns	0.8254
45	-0.7571	-2.270 to 0.7555	ns	0.9993
50	-1.605	-3.117 to -0.09215	*	**0.0232**
55	-0.231	-1.744 to 1.282	ns	>0.9999
60	-1.448	-2.960 to 0.06499	ns	0.0832
65	-0.6595	-2.172 to 0.8531	ns	>0.9999
70	-0.681	-2.194 to 0.8317	ns	>0.9999
75	-1.545	-3.058 to -0.03263	*	**0.0383**
80	-0.4	-1.913 to 1.113	ns	>0.9999
85	-1.248	-2.760 to 0.2650	ns	0.3217
90	-1.107	-2.620 to 0.4055	ns	0.6307
95	-1.652	-3.165 to -0.1398	*	**0.0153**
100	-1.252	-2.765 to 0.2602	ns	0.313
105	-1.612	-3.125 to -0.09929	*	**0.0218**
110	-1.357	-2.870 to 0.1555	ns	**0.1605**
115	-1.231	-2.744 to 0.2817	ns	0.3535
120	-0.95	-2.463 to 0.5626	ns	0.9243
125	-1.207	-2.720 to 0.3055	ns	0.4019
130	-1.126	-2.639 to 0.3864	ns	0.5857

135	-0.7333	-2.246 to 0.7793	ns	0.9997
140	-0.8286	-2.341 to 0.6840	ns	0.9934
145	-0.5333	-2.046 to 0.9793	ns	>0.9999
150	-0.5762	-2.089 to 0.9364	ns	>0.9999
155	-0.2381	-1.751 to 1.275	ns	>0.9999
160	-0.03571	-1.548 to 1.477	ns	>0.9999
165	0.02143	-1.491 to 1.534	ns	>0.9999
170	-0.01429	-1.527 to 1.498	ns	>0.9999
175	0.2905	-1.222 to 1.803	ns	>0.9999
180	0.1905	-1.322 to 1.703	ns	>0.9999
185	0.08333	-1.429 to 1.596	ns	>0.9999
190	0.1286	-1.384 to 1.641	ns	>0.9999
195	0.05476	-1.458 to 1.567	ns	>0.9999
200	0.2048	-1.308 to 1.717	ns	>0.9999
205	0.1071	-1.405 to 1.620	ns	>0.9999
210	0.1238	-1.389 to 1.636	ns	>0.9999
215	0.1048	-1.408 to 1.617	ns	>0.9999
220	0.1214	-1.391 to 1.634	ns	>0.9999
225	0.1333	-1.379 to 1.646	ns	>0.9999
230	-0.02381	-1.536 to 1.489	ns	>0.9999
235	0.04762	-1.465 to 1.560	ns	>0.9999
240	-0.01429	-1.527 to 1.498	ns	>0.9999
245	-0.01429	-1.527 to 1.498	ns	>0.9999
250	-0.181	-1.694 to 1.332	ns	>0.9999
255	-0.007143	-1.520 to 1.505	ns	>0.9999
260	-0.1786	-1.691 to 1.334	ns	>0.9999
265	-0.2857	-1.798 to 1.227	ns	>0.9999
270	-0.2952	-1.808 to 1.217	ns	>0.9999
275	-0.3048	-1.817 to 1.208	ns	>0.9999
280	-0.281	-1.794 to 1.232	ns	>0.9999
285	-0.1881	-1.701 to 1.325	ns	>0.9999
290	-0.1024	-1.615 to 1.410	ns	>0.9999
295	-0.119	-1.632 to 1.394	ns	>0.9999
300	-0.03571	-1.548 to 1.477	ns	>0.9999
305	-0.01667	-1.529 to 1.496	ns	>0.9999
310	0.1095	-1.403 to 1.622	ns	>0.9999
315	0.119	-1.394 to 1.632	ns	>0.9999
320	0.2119	-1.301 to 1.725	ns	>0.9999
325	0.169	-1.344 to 1.682	ns	>0.9999
330	0.2643	-1.248 to 1.777	ns	>0.9999

335	0.181	-1.332 to 1.694	ns	>0.9999
340	0.2429	-1.270 to 1.755	ns	>0.9999
345	0.0619	-1.451 to 1.575	ns	>0.9999
350	-0.0119	-1.525 to 1.501	ns	>0.9999
355	-0.01667	-1.529 to 1.496	ns	>0.9999
360	-0.01905	-1.532 to 1.494	ns	>0.9999
365	-0.02381	-1.536 to 1.489	ns	>0.9999
370	0.009524	-1.503 to 1.522	ns	>0.9999
375	-0.02619	-1.539 to 1.486	ns	>0.9999
380	-0.02619	-1.539 to 1.486	ns	>0.9999
385	-0.09762	-1.610 to 1.415	ns	>0.9999
390	-0.09762	-1.610 to 1.415	ns	>0.9999
395	-0.03095	-1.544 to 1.482	ns	>0.9999
400	0.002381	-1.510 to 1.515	ns	>0.9999

Table S3. List of all genes (n=139) found to be differentially expressed in *Phf21b*$^{+/+}$ and *Phf21b*$^{\Delta4/\Delta4}$ hippocampal tissues, with their respective fold changes, p-values, and false discovery rates. RNAseq data were deposited under the accession number GSE201477, NCBI GEO repository.

Gene ID	Fold change	P-value	False discovery rate (FDR)
Gm5741	-3247.63	0.00015	0.058641
Irx2	-493.272	8.69E-07	0.004523
Pou4f1	-340.463	1.25E-06	0.004523
Chat	-255.711	1.20E-05	0.015308
D130079A08Rik	-230.363	0.000223	0.064576
Slc18a3	-136.325	8.48E-05	0.043867
Shox2	-133.375	0.000527	0.078629
D130009I18Rik	-130.756	1.88E-05	0.01675
Gm38534	-89.0792	9.75E-06	0.015308
Nppa	-80.2295	1.97E-05	0.01675
Chrnb4	-52.218	1.27E-05	0.015308
Chrna3	-42.4243	9.45E-07	0.004523
Gpr151	-34.6766	2.38E-07	0.003446
Wfikkn2	-28.6555	0.000178	0.061679
Irx1	-22.8385	0.000199	0.061679
Irx5	-20.6858	0.000188	0.061679
Cytip	-16.9811	4.74E-05	0.028581
Slc5a7	-15.5758	1.74E-05	0.01675
Gng8	-15.5672	1.13E-05	0.015308
Tyrp1	-14.57	0.000129	0.053711

Tmem182	-13.7779	2.29E-05	0.018439
Tafa4	-13.1165	1.08E-05	0.015308
Gm13373	-11.9992	3.49E-05	0.023739
Tac2	-11.3515	3.79E-06	0.009145
Cubn	-10.6913	3.67E-05	0.023739
Chrnb3	-9.74694	0.000319	0.068212
Irx3	-9.4892	0.000329	0.068212
Syt9	-9.17583	0.000496	0.075841
Nhlh2	-9.03027	8.93E-06	0.015308
Tcf7l2	-8.68022	3.39E-05	0.023739
Avil	-8.63494	0.000259	0.068176
Crabp2	-8.29422	0.000771	0.092211
Syt15	-8.05554	0.000124	0.053711
Sncg	-6.55309	1.78E-05	0.01675
Cldn19	-6.27332	0.000316	0.068212
Ano1	-6.17137	1.96E-06	0.005679
Ascl4	-6.07173	0.00054	0.078629
Pla2r1	-5.50737	0.000268	0.068207
Scx	-5.30418	0.000319	0.068212
Pld5	-5.23611	0.000731	0.089708
Syt6	-5.16257	0.000104	0.050138
Lrrc55	-5.06769	0.000379	0.071256
Fzd10	-5.02458	0.000171	0.061679
Wif1	-4.5528	0.000198	0.061679
Cftr	-4.53162	0.000715	0.088883

Cnmd	-4.34976	0.000781	0.092451
Vav3	-4.26307	0.000156	0.059417
Npr1	-4.25244	0.000188	0.061679
Kcng4	-4.03663	0.000203	0.061679
Spx	-3.99011	0.000377	0.071256
Kcnh8	-3.9395	0.000858	0.094412
Gpr179	-3.93778	0.000457	0.075246
Adcyap1	-3.68078	0.000557	0.079838
Scn9a	-3.56366	0.000241	0.067738
Tex15	-3.17861	0.000258	0.068176
Cdca7l	-3.17687	0.000407	0.072325
Amotl1	-2.93533	0.000371	0.071256
Aebp1	-2.75947	0.000247	0.067738
Ptgds	-2.63231	6.39E-05	0.035566
Cep112	-2.56008	0.00052	0.07843
Gpr153	-2.47073	0.000662	0.086294
Prkch	-2.32653	0.000543	0.078629
Wls	-2.1938	0.000442	0.073622
Rflnb	-2.02805	0.000861	0.094412
Gm50431	-2.01919	0.000292	0.068207
Hhip	-1.97454	0.00033	0.068212
Cdyl	-1.94559	0.000248	0.067738
Id3	-1.93144	0.00032	0.068212
Fzd1	-1.88878	0.000598	0.083266
Lca5	-1.82995	0.000819	0.092575

Cnnm2	-1.76155	8.98E-05	0.044807
Isoc1	-1.74687	0.000677	0.086798
Slc29a1	-1.6707	0.000309	0.068212
Pdzd2	-1.61176	0.000417	0.072325
Aqp4	-1.59437	0.000405	0.072325
Shisal1	-1.55267	0.000653	0.086001
Axin2	-1.51971	0.000176	0.061679
Arhgap24	-1.50305	0.000787	0.092451
Nek7	-1.46064	0.000564	0.08003
Rpl39	-1.317	0.000884	0.094781
Adgrb3	1.360114	0.000917	0.097078
Cdh11	1.369075	0.000577	0.081114
Sel1l	1.419391	0.000403	0.072325
Csrnp2	1.424667	0.000617	0.083948
Cdh10	1.428154	0.000205	0.061679
Usp46	1.455037	0.000753	0.091154
Pld3	1.455462	0.000478	0.075841
Large1	1.460375	0.000835	0.09349
Tmem74	1.471975	0.000919	0.097078
Homer1	1.488299	0.000282	0.068207
Rap2b	1.493232	0.000286	0.068207
Lrrn2	1.499845	0.000498	0.075841
Adam23	1.508529	0.000711	0.088883
St8sia3	1.527343	0.000277	0.068207
Gad2	1.541043	0.000756	0.091154

Brinp2	1.599715	7.21E-05	0.038658
Ppm1e	1.608113	0.000494	0.075841
Pls3	1.610765	0.000374	0.071256
Ext1	1.615763	0.000482	0.075841
Ap2b1	1.620352	0.00071	0.088883
Mal2	1.637022	0.000386	0.071598
Pcdhga2	1.649238	0.000951	0.09907
Klhl3	1.658838	0.000814	0.092575
Sertm1	1.675461	0.000128	0.053711
Prkcb	1.678694	0.000882	0.094781
Nr4a1	1.688614	6.29E-05	0.035566
Rasl11b	1.696932	0.000127	0.053711
Sept9	1.702277	0.00013	0.053711
Zdhhc23	1.704707	0.000485	0.075841
Synj2	1.727667	0.000434	0.073622
Plxna1	1.748143	0.00042	0.072325
Htr2a	1.757264	0.000638	0.084698
Tmem121b	1.765785	0.000718	0.088883
Sh3rf1	1.772809	0.000196	0.061679
Cacna2d3	1.777189	0.000473	0.075841
Tmem200a	1.802365	0.00067	0.086551
Crhbp	1.821505	3.77E-05	0.023739
Akap5	1.834138	0.000325	0.068212
Sstr4	1.834562	0.000815	0.092575
Ar	1.836513	0.000441	0.073622

Vgf	1.882551	0.000947	0.09907
Mchr1	1.887289	0.000807	0.092575
Sorcs3	1.919584	1.44E-05	0.016027
Dlx1as	1.927339	0.000881	0.094781
Camk4	1.929184	0.000792	0.092451
Galntl6	1.962446	0.000272	0.068207
Fam189a1	1.993603	0.000347	0.06966
Htr1a	2.020346	0.000149	0.058641
Foxp1	2.090582	0.000336	0.068554
Lancl3	2.104651	0.000629	0.084286
Vxn	2.186427	0.00084	0.09349
Galnt9	2.205406	0.000351	0.06966
Fyb2	2.359708	0.000215	0.063635
Gpr161	2.53297	3.58E-05	0.023739
Hcn1	2.593056	0.00062	0.083948
Gm10754	2.604448	0.000539	0.078629
Mpped1	2.70499	0.000608	0.08377
Fibcd1	2.921538	0.000289	0.068207
Ptpru	2.977622	0.000413	0.072325

Table S4. Sequences of primers used in qRT-PCR experiments.

Target	Forward sequence (5′ → 3′)	Reverse sequence (5′ → 3′)
Chat (mRNA)	CCAATGACCAGCTAAGGTTTG	CAGTCAGTGGGAATGGATTGG
Fibcd1	ACAAACCGCAGCGAGCAAG	TTCAGGAAGAGAACCACACCG
Gapdh	ATGCCATCACTGCCACCCAGAAG	TGCCAGTGAGCTTCCCGTTCAG
Gm5741	GGCATCAACCGCATAAAGGTATC	ACAAGGAAGGGGTCGCTCTT
Kdm1a	GGTTGTAACAGGTCTTGGAGGG	GGAACAGCTTGTCCATTGGC
Nppa	CGTCTTGGCCTTTTGGCTTC	GTGGTCTAGCAGGTTCTTGAAA
Phf21a	TCTAACTGCATCACAGAAGACTG	AGCAATGGTAGGTCTCTGGC
Phf21b Primer 1 (Exon 1-2)	GCCAAAGTGAGCGCAAAGTG	GAGGTCGCCGTTCTGGTG
Phf21b Primer 2 (Exon 11-12)	CAGCGGAAGGCCTTAAAGAA	CACTGTCTTGTGGGTGACATAG
Slc18a3	AGACTATGCCACGCTCTTCG	AGGCTCCTCGGGATACTTGT
Scn1a	TTCAGGGGCTATCGAGGCTG	TTCATGTGAGATTCCCCCGAAA
Shank3	GGACCTGCAACAAACGAAGT	GGCTGGAATAGCCCGTAGTT
Vav3	CGGCGCAGGTGTTCGAC	GTTCTTCAAACAGAGAAACTGGGAC
Chat (TSS)	TGGCGTCGCTCATGCTAATA	GGAAACATCTCGGAAGCGGA
Chat (-1000 bp)	TAACAGTGCCAAGGGAGACG	TGCGTGTACCACACCCTAAG
Chat (-2000 bp)	TCGCGCCGTCTGAGTAAAAT	AGAGAAAACTGCTGCCTCCC
Chat (-3000bp)	ATGGTCTCAGGGCTCAATGC	AAAACCCACCACCCACTCTG
Chat (+1000 bp)	CCAAGTAGGCGCTGGCATTA	GTGCCCATTGTTCCCGACTA

Chat (+2000 bp)	AGGAGTAGGAGTGCGTCGAA	GGGGCATCCTCTACGAGTTC
Chat (+3000 bp)	GAGTGTGTTTGGAGAGGGGG	GGAATCCCTTCTCCGTGACC
Grin2b	GCCATGAACGAGACTGACCC	GCTTCCTGGTCCGTGTCATC
Gria1	ACCCTCCATGTGATCGAAATG	GGTTCTATTCTGGACGCTTGAG

References

1. Bae S, Park J, and Kim JS. Cas-OFFinder: a fast and versatile algorithm that searches for potential off-target sites of Cas9 RNA-guided endonucleases. *Bioinformatics.* 2014;30(10):1473-5.
2. Can A, Dao DT, Terrillion CE, Piantadosi SC, Bhat S, and Gould TD. The tail suspension test. *J Vis Exp.* 2012(59):e3769.
3. Can A, Dao DT, Arad M, Terrillion CE, Piantadosi SC, and Gould TD. The mouse forced swim test. *J Vis Exp.* 2012(59):e3638.
4. Vorhees CV, and Williams MT. Morris water maze: procedures for assessing spatial and related forms of learning and memory. *Nat Protoc.* 2006;1(2):848-58.
5. Chin EWM, and Goh ELK. Behavioral Characterization of MeCP2 Dysfunction-Associated Rett Syndrome and Neuropsychiatric Disorders. *Methods Mol Biol.* 2019;2011:593-605.
6. Thor DH, and Holloway WR. Social memory of the male laboratory rat. *J Comp Physiol Psychol.* 1982;96(6):1000-6.
7. Hitti FL, and Siegelbaum SA. The hippocampal CA2 region is essential for social memory. *Nature.* 2014;508(7494):88-92.
8. Yang M, and Crawley JN. Simple behavioral assessment of mouse olfaction. *Curr Protoc Neurosci.* 2009;Chapter 8:Unit 8 24.
